# Resistance to Targeted Therapy in AML: Current Challenges and Emerging Treatment Strategies

**DOI:** 10.3390/jcm15062171

**Published:** 2026-03-12

**Authors:** Christos Stafylidis, Panagiotis T. Diamantopoulos

**Affiliations:** Hematology Unit, First Department of Internal Medicine, National and Kapodistrian University of Athens, Laikon General Hospital, 115 27 Athens, Greece; stafi123@hotmail.com

**Keywords:** acute myeloid leukemia, BCL-2 inhibitors, venetoclax, FLT3 inhibitors, IDH1 inhibitors, IDH2 inhibitors, menin inhibitors

## Abstract

The development of targeted treatments, including inhibitors of BCL-2, FLT3, IDH1/2, and menin, has significantly expanded the therapeutic landscape of acute myeloid leukemia (AML), offering more personalized and molecularly driven treatment approaches. Despite these advances, achieving durable responses represents a major challenge, limited by the emergence of intrinsic and acquired resistance to targeted agents. This review summarizes the current understanding of the cellular and molecular mechanisms underlying resistance to targeted therapies in AML. Key mechanisms include acquired mutations that alter the drug target, other co-occurring genetic and epigenetic alterations, activation of bypass signaling pathways, and metabolic reprogramming. Furthermore, the role of clonal heterogeneity and the bone marrow microenvironment in the development of resistance is increasingly recognized. In addition, we discuss emerging strategies aiming at overcoming resistance, such as combination treatments and novel inhibitors designed to target resistant clones. Finally, this review highlights the critical need for mechanism-driven therapeutic design in order to achieve sustained responses and improve long-term outcomes in patients with AML.

## 1. Introduction

Recent advances in understanding the molecular and genetic landscape of AML have enabled more refined prognostic assessment and have fundamentally reshaped its therapeutic approach [1,2]. The identification of recurrent genomic abnormalities implicated in AML pathogenesis has facilitated the development of targeted agents, paving the way for more personalized treatment strategies, particularly for patients ineligible for intensive chemotherapy (IC) or those who relapse after standard therapy [2].

Targeted therapy is now a key component of AML treatment, guided by molecular profiling at diagnosis. Currently approved targeted agents include inhibitors of B-cell lymphoma 2 (BCL-2), fms-like tyrosine kinase 3 (FLT3), isocitrate dehydrogenase (IDH), and, more recently, menin, while additional agents targeting other genomic alterations, such as TP53 mutations, are currently under investigation [2,3]. Although targeted therapy can yield meaningful clinical outcomes, its benefit is limited by the emergence of resistance, with most patients ultimately developing refractoriness or experiencing relapse [4,5,6,7].

In this review, we summarize the currently approved targeted agents for AML treatment and critically examine the molecular mechanisms underlying intrinsic or acquired resistance. In addition, we discuss emerging strategies aimed at overcoming resistance and improving outcomes with targeted therapies.

## 2. BCL-2 Inhibitors

BCL-2 is a key anti-apoptotic protein, located in the outer mitochondrial membrane. It regulates the mitochondrial apoptotic pathway by binding the pro-apoptotic BAX/BAK proteins, subsequently precluding mitochondrial outer membrane permeabilization (MOMP) and the release of cytochrome c, ultimately leading to the prevention of apoptosis [8,9].

BCL-2 is overexpressed in several B-cell malignancies, such as follicular lymphoma, chronic lymphocytic leukemia (CLL), and diffuse large B-cell lymphoma, highlighting the apoptosis dysregulation associated with these disorders [10,11,12]. Moreover, aberrant expression of BCL-2 has been found to confer resistance of AML cells to standard chemotherapy [13].

Venetoclax (VCT/ABT-199/VEN) is an oral, highly selective BCL-2 inhibitor (BCL-2i) that, mimicking the BH3 domain and binding the BCL-2 protein, induces the release of pro-apoptotic proteins. VEN has been approved for treatment-naïve and relapsed/refractory (R/R) CLL as well as for newly diagnosed (ND) AML in patients unfit for IC, in combination with hypomethylating agents (HMAs) [14,15]. Efficacy of VEN combined with azacitidine (AZA) has been established in pivotal clinical trials [16,17].

Despite the elevated BCL-2 expression in AML cells, the use of VEN as monotherapy in AML has resulted in modest therapeutic outcomes, suggesting inherent resistance to the drug [18]. Furthermore, the combination of AZA and VEN yielded no initial response in 27% of AML patients, while the majority of initial responders eventually relapsed [17]. These findings imply the presence of both intrinsic and acquired resistance to VEN.

### 2.1. Mechanisms Contributing to Resistance to BCL-2 Inhibitors

**Figure 1:**
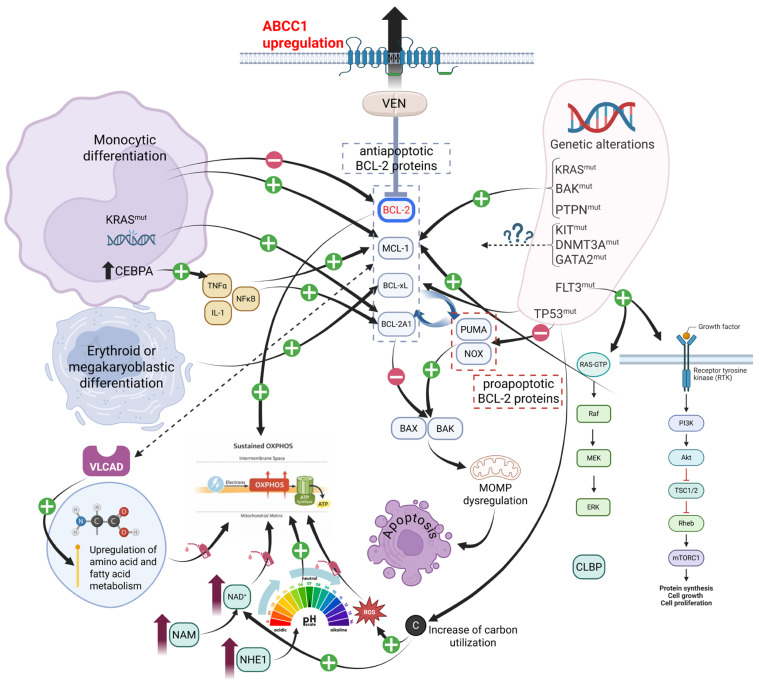
Mechanisms underlying resistance to venetoclax involving regulation of apoptosis, genetic alterations and metabolic rewiring. This figure summarizes the major molecular and cellular mechanisms contributing to the development of resistance to the BCL-2 inhibitor venetoclax (VEN). As seen at the center, resistance emerges through compensatory upregulation of alternative anti-apoptotic BCL-2 family members, including MCL-1, BCL-xL, and BCL-2A1, that prevent BAX/BAK activation, mitochondrial outer membrane permeabilization (MOMP), and ultimately apoptosis. Reduced intracellular drug availability due to ABCC1 upregulation (upper middle) further limits VEN efficacy. Lineage differentiation state (upper left) also affects sensitivity to VEN. Monocytic differentiation is correlated with downregulation of BCL-2 and upregulation of MCL-1. Moreover, it is frequently associated with KRAS mutations as well as activation of inflammatory signaling (TNFα/IL-1/NFκB) due to increased CEBPA expression, which further result in MCL-1 and BCL-2A1 upregulation. Similarly, erythroid or megakaryocytic differentiation (middle left) is linked with upregulation of BCL-xL. Genetic alterations, such as BAK, KRAS, PTPN, KIT, DNMT3A, GATA2, FLT3, and TP53 mutations (upper right), also lead to altered anti-apoptotic protein expression and promote apoptotic escape. TP53 mutations also impair the induction of pro-apoptotic proteins PUMA and NOXA, thereby attenuating mitochondrial apoptosis, whereas FLT3 mutations further promote survival signaling through activation of RAF-MEK-ERK and PI3K-AKT-Mtor (middle right). Metabolic rewiring (lower left) supports resistance through sustained oxidative phosphorylation (OXPHOS), which is normally inhibited by BCL-2 inhibition, driven by upregulation of fatty acid and amino acid metabolism, and increased through NAD+ generation, carbon utilization induced by TP53 mutations, and intracellular pH induced by Na+/H+ (NHE1) exchanger 1 activation. Finally, the upregulation of CLBP and OPA-1 which modulates mitochondrial cristae and mitofusin-2 (MFN2) and subsequently regulates mitochondrial autophagy (lower right) leads to alterations in mitochondrial integrity and impairment of MOMP. Created in BioRender. Panagiotis T. Diamantopoulos. (2026). https://BioRender.com/.

Mechanisms underlying intrinsic or acquired resistance to VEN in AML are multifactorial, as shown in Figure 1, and can be broadly categorized as follows.

#### 2.1.1. Shifts in Anti-Apoptotic BCL-2 Family Dependency

One of the most important factors contributing to altered susceptibility to BCL-2 inhibition is the upregulation of alternative anti-apoptotic BCL-2 family members, including BCL-xL, myeloid cell leukemia 1 (MCL-1), and BCL-2-related protein A1 (BCL-2A1). Preclinical studies have demonstrated elevated MCL-1 and BCL-xL and decreased BCL-2 levels in VEN-resistant AML cell lines [19,20]. Notably, restoration of VEN sensitivity was observed by inhibiting MCL-1 and BCL-xL [19]. Importantly, a shorter duration of VEN responses was recorded in patients with MCL-1 or BCL-xL dependence by using BH3 profiling in a phase 2 study of AML patients treated with VEN monotherapy [18]. Similarly, upregulation of BCL-2A1 has been correlated with VEN resistance. Contrary to lymphoproliferative disorders, where BCL-xL primarily contributes to VEN resistance [5], MCL-1 seems to be the key contributor to VEN resistance in AML, since it is the most highly expressed anti-apoptotic BCL-2 family member in AML cells [21]. Furthermore, the blockade of MCL-1 in a murine AML model resulted in a significant reduction in leukemic cells in vivo, while disease relapse occurred only in mice bearing cells that retained MCL-1 expression, thus further supporting the importance of MCL-1 dependency in AML [22]. Finally, acquired overexpression of MCL-1 has been observed in AML cells with chronic exposure to VEN [19]. Collectively, this data suggests that resistance to VEN is primarily mediated by the intrinsic dependency of AML cells on MCL-1 and/or other anti-apoptotic proteins and is dynamically increased via further elevation of MCL-1.

#### 2.1.2. Lineage-Specific Anti-Apoptotic Dependencies

Recent studies have indicated that VEN exhibits significantly variable efficacy across AML subtypes. In particular, AML with monocytic differentiation displays intrinsic resistance to VEN treatment, whereas monocytic subclones also confer resistance to VEN treatment. An ex vivo drug sensitivity testing on AML patient samples has shown a continuous increase in VEN resistance from the undifferentiated AML (M0) to monocytic AML (M5) [23]. Additionally, a higher expression of CD14 and CLEA7A, which is usually found in M4/M5 AML, has been associated with VEN resistance [24].

These findings may be attributed to several factors. A progressive decline in BCL-2 expression, accompanied by a concurrent increase in MCL-1 expression, has been observed from M0 to M5 [24,25]. Moreover, the presence of *KRAS* mutations and elevated BCL-2A1 expression, which confer resistance to VEN, has also been reported in M4/M5 AML [22,24]. Additionally, monocytic AML has a distinct transcriptomic profile and relies more on MCL-1 than BCL-2 to mediate oxidative phosphorylation and survival [25]. It has been shown that elevated levels of CCAAT enhancer-binding protein B (CEBPB) underlie VEN resistance in monocytic AML [26]. CEBPB is highly expressed in monocytic AML cells and induces upregulation of MCL-1, BCL-2A1, and the interleukin-1 (IL-1)/tumor necrosis factor alpha (TNF-α)/NF-κB pathway members, whereas IL-1 and TNF-α specifically increase CEBPB levels and protect M4/M5 from VEN, but not M0/M1 leukemia cells [26]. Hence, this CEBPB-IL-1/TNF-a monocyte differentiation positive feedback loop promotes both intrinsic and extrinsic drug resistance in M4/M5 leukemia.

Sensitivity to VEN is strongly influenced by developmental heterogeneity within the leukemic clone [25]. Distinct genetic subclones can coexist at variable differentiation stages, with monocytic differentiation intrinsically contributing to VEN resistance. Analyses of patient samples have revealed that VEN successfully eliminated primitive leukemia stem cell (LSC) populations but allowed the expansion of resistant monocytic subclones while retaining MCL-1 dependency [25]. Finally, the co-existence of primitive and monocytic LSC populations within individual patients may drive intrinsic and acquired resistance.

Resistance to VEN has also been reported in AML with erythroid or megakaryocytic differentiation, since those cells depend mostly on BCL-xL, rather than BCL-2 [27]. Studies in cell lines and animal models have shown diminished efficacy of BCL-2i, while BCL-xL inhibition has yielded promising efficacy [27].

#### 2.1.3. Metabolic Reprogramming and Mitochondrial Integrity

Alterations in mitochondrial metabolism, as well as other metabolic adaptations, have emerged as important mechanisms underlying the development of VEN resistance. Mitochondria are essential for regulating cellular respiration and apoptosis, while BCL-2 family members have been found to regulate enzymes of various mitochondrial metabolic pathways [28].

AML cells are highly dependent on oxidative phosphorylation (OXPHOS) for energy production [28]. Interestingly, BCL-2 supports OXPHOS, particularly in quiescent LSCs [28]. VEN, combined with AZA, has been shown to target LSC metabolism by decreasing amino acid uptake in these cells and by inhibiting electron transfer complex (ETC) II activity [29]. Interestingly, VEN-induced metabolic reprogramming is independent of BCL-2 inhibition [30]. However, AML cells frequently undergo metabolic reprogramming by shifting to energy generation from alternative metabolic pathways, which results in resistance [31,32,33].

A shift towards fatty acid metabolism and upregulation of fatty acid oxidation (FAO) has been observed in patients resistant to VEN [31]. These alterations occur as a compensatory adaptation in relapsed disease [31]. Furthermore, MCL-1, which is frequently upregulated in VEN-resistant cells, directly interacts with very long-chain acyl-CoA dehydrogenase (VLCAD), which is an enzyme of the mitochondrial fatty acid β-oxidation pathway [32]. Knockdown of VLCAD has resulted in restoration of sensitivity to VEN, while similar results have also been observed upon MCL-1 inhibition [31]. Inhibition of fatty acid mitochondrial transporters CPT1A and CPT1C has also led to overcoming VEN resistance [31].

Elevated levels of nicotinamide and subsequently increased levels of nicotinamide adenine dinucleotide (NAD+) have been found in relapsed LSCs [33]. In particular, LSCs rely on increased NAD+ levels, which activate both amino acid metabolism and FAO, to raise the flux of amino acids and fatty acids into the tricarboxylic acid (TCA) cycle and eventually sustain OXPHOS [33]. Finally, overexpression of the ATP-binding cassette transporter 1 (ABCC1) and enhanced glutathione metabolism mediate VEN resistance by reducing intracellular VEN levels, thereby limiting its cytotoxicity [34].

Metabolomic studies have revealed that activation of the RAS/MAPK pathway induces upregulation of MCL-1 and increases FAO and amino acid metabolism, thereby providing alternative fuels to sustain OXPHOS [35]. Hence, activation of the RAS/MAPK pathway augments the metabolic plasticity of leukemic cells, thus mediating VEN resistance. Similarly, activation of the PI3K-AKT-mTOR pathway has been shown to regulate metabolic flux that contributes to VEN resistance [36]. In more detail, redox-mediated AKT activation stabilizes MCL-1, contributing to resistance [37]. Finally, a recent study has indicated that Na^+^/H^+^ exchanger 1 (NHE1) activation confers resistance to VEN by increasing intracellular pH and activating PI3K/Akt, ultimately leading to MCL-1 upregulation [38].

The efficacy of VEN indirectly relies on its ability to induce MOMP; hence, mechanisms that maintain mitochondrial structural integrity, such as regulation of cristae architecture and/or their interaction with other organelles, also play a central role in VEN adaptive resistance. Mitochondrial reprogramming has also been recognized as a factor of VEN resistance in lymphoid malignancies [39]. Caseinolytic peptidase B protein homolog (CLBP) is an ATPase associated with diverse cellular activities (AAA+ ATPase), serving as a protein chaperone. It modulates mitochondrial cristae morphology, possibly by interacting with optic atrophy protein type 1 (OPA-1) and preventing its proteolytic cleavage [40]. OPA-1 is a protein located in the inner mitochondrial membrane undergoing constitutive proteolytic processing, which is essential for preserving mitochondrial cristae. VEN-resistant AML cells upregulate OPA-1, rendering the mitochondrial cristae tighter, thus evading apoptosis [40]. Furthermore, CLBP is highly expressed in AML cells and is further upregulated upon acquisition of VEN resistance. Importantly, depletion of CLPB re-sensitizes cells to VEN [40]. Further research has shown that elevated levels of mitofusin-2 (MFN2), a regulator of mitochondrial autophagy (mitophagy), have been correlated with VEN resistance [41]. Interestingly, resistance to BCL-2 inhibition is accompanied by enhanced mitochondria–endoplasmic reticulum interactions and enhanced mitophagy flux [41].

#### 2.1.4. Genetic Alterations

Genetic alterations are also implicated in the development of resistance to BCL-2i. A recent classification has stratified AML patients treated with AZA and VEN into three prognostic groups, which are primarily defined by the presence or absence of *FLT3*-internal tandem duplication (*FLT3*-ITD), *K/NRAS*, and *TP53* mutations [1].

Genomic instability is more prominent in CLL patients who frequently bear *BCL2* mutations, such as Gly101Val, which confer resistance to VEN treatment [42]. Notably, *BCL2* mutations have not been reported in AML. However, acquired inactivating missense or frameshift/nonsense BAX mutations are a novel mechanism of adaptive resistance, since they have been observed in 17% of AML patients who relapsed after receiving VEN-based regimens [43].

Mutations in *TP53* can be found in 5–10% of de novo AML patients, and multiple clinical trials have associated *TP53* abnormalities with resistance to VEN treatment [3,44,45]. Previous studies have demonstrated that *TP53*-mutated AML patients display inferior responses and relapse early after VEN treatment, despite occasional initial remissions [46]. In detail, loss of *TP53* elevates the apoptotic threshold, rather than abrogating sensitivity to VEN, allowing leukemic cells to even at optimal drug exposure [47,48]. Moreover, several pro-apoptotic genes involved in the mitochondrial apoptosis pathway, including the BH3-only proteins PUMA and NOXA and the effector proteins BAK and BAX, are transcriptionally regulated by TP53 [49]. In *TP53*-knockout AML cells, the reduced expression and delayed activation of BAX and BAK impair MOMP, thus attenuating VEN-induced apoptosis [47,48]. Besides its direct apoptotic defects, inactivation of *TP53* prompts mitochondrial and metabolic adaptations, including altered mitophagy, increased reactive oxygen species, and reprogrammed carbon utilization with increased nucleotide biosynthesis [48]. Finally, loss of *TP53* has also been correlated with an increased BCL-xL/BCL-2 ratio, further decreasing VEN efficacy [48].

Mutations in activating kinase genes, including *FLT3*-ITD and *KRAS/PTPN1*, have also been associated with VEN resistance [50]. *FLT3*-ITD mutations enhance survival by activating various intracellular signaling pathways, including PI3K-Akt, RAS-MAPK, and STAT5, thereby leading to increased expression of BCL-xL and MCL-1 [51,52]. Previous research has shown that AML cells harboring *KRAS* mutations have low BCL-2 and BAX levels and high MCL-1 and BCL-2A1 levels [24]. It has also been suggested that *KRAS* mutations depend only on MCL-1 to drive VEN resistance, while *PTPN1* mutations are partially dependent on MCL-1 and BCL-xL [24]. Finally, besides mutations in activating kinases, several other mutations, such as in *KIT*, *DNMT3A*, and *GATA2*, have also been implicated [53,54].

Interestingly, several genetic alterations have been linked to heightened sensitivity to VEN. Mutations in *IDH1* and *IDH2* lead to the accumulation of the oncometabolite 2-hydroxyglutarate (2-HG), which impairs mitochondrial respiration and lowers the apoptotic threshold by increasing cellular dependency on BCL-2 [55]. Moreover, mutations in spliceosome components, including *SRSF2*, *U2AF1*, *SF3B1*, and *ZRSR2*, also favor BCL-2 reliance through alterations in the splicing and expression of apoptosis-related genes [56]. Additionally, *ASXL1* mutations induce BCL-2 overexpression by increasing chromatin accessibility to its promoter, driven by defective PRC2-mediated repression [57]. Finally, *NPM1* mutations have been associated with enhanced responses to VEN [50]. Although the exact mechanism remains unknown, increased VEN sensitivity may be explained by the fact that *NPM1* mutations induce the overexpression of homeobox genes associated with sensitivity to BCL-2 inhibition in AML cells [58,59].

### 2.2. Strategies to Overcome Venetoclax Resistance

An improved understanding of the mechanisms underlying venetoclax resistance over recent years has led to the development of several strategies aimed at overcoming these challenges. Key strategies incorporating venetoclax-based combination regimens are summarized in Table 1 and discussed below.

#### 2.2.1. Targeting MCL-1 and Other Apoptotic Proteins

Given that altered apoptotic protein expression is a key mechanism of venetoclax resistance, combining venetoclax with inhibitors targeting other BCL-2 family members represents a rational therapeutic strategy. The clinical use of navitoclax, an early BH3 mimetic that also targets BCL-xL in addition to BCL-2, has been confined due to on-target thrombocytopenia, since platelet survival relies on BCL-xL [60]. However, a phase 1 clinical trial combining navitoclax with VEN and DEC in AML patients previously treated with VEN is ongoing [61]. Interestingly, AZD4320, a novel dual BCL-2/BCL-xL inhibitor, has proven its efficacy in patient-derived AML and VEN-resistant xenograft models with only limited platelet toxicity [62]. Unfortunately, AZD0466, a drug–dendrimer conjugate exerting dual BCL-2/BCL-xL inhibition, has failed to elicit significant responses in a phase 1/2 trial in AML (NCT04865419) [63]. Notably, lisaftoclax (APG-2575), a novel BCL-2i, combined with AZA has displayed encouraging efficacy in R/R AML, including patients refractory to VEN [64].

a. Direct MCL-1 inhibitors

As discussed earlier, MCL-1 is an attractive target prompting the development of direct MCL-1 inhibitors [22]. S63845 was the first MCL-1 inhibitor to be developed and has proved its efficacy in vitro and in xenograft mouse models [65,66]. S63845 successfully eliminates AML cells but also induces profound myelosuppression, thus deterring its entry into clinical trials [65,66]. S64315 (MIK655), another MCL-1 inhibitor, has progressed into a phase 1/2 clinical trial in combination with AZA for AML (NCT04629443) [67]. MIK655 was also investigated in R/R AML in combination with VOB560, a novel BCL-2i, and despite the reported encouraging results, recruitment was halted [68]. After proving their in vitro efficacy, AMG-176 (tapotoclax) and AMG-397 (murizatoclax) entered phase 1 clinical trials either as monotherapy (NCT02675452) or as combined treatment (NCT05209152, NCT03797261, NCT03465540) for patients with R/R AML, but were prematurely terminated due to increased cardiotoxicity, which was considered an on-target effect of MCL-1 inhibition [69]. Likewise, increased cardiac toxicity was observed with AZD5991, which has shown promising efficacy when combined with VEN in mouse xenograft studies of human AML [70]. This resulted in early suspension of a clinical trial of AZD5991 in combination with VEN in R/R AML (NCT03218683) [71]. Another selective MCL-1 inhibitor with proven in vitro and in vivo efficacy is PRT1419 and is being investigated in clinical trials (NCT05107856, NCT04543305). Overall, these results suggest that while direct MCL-1 inhibitors are highly promising, careful attention to dose-limiting toxicities, particularly myelosuppression and cardiotoxicity, is essential to enable successful clinical development.

b. Indirect MCL-1 inhibition

Several agents that indirectly induce downregulation of MCL-1 have been investigated in AML patients. The combination of HMAs with VEN has been based on the synergistic effects exerted by these agents [29,72]. Although the exact synergistic mechanisms remain unknown, it has been demonstrated that AZA downregulates MCL-1 and upregulates pro-apoptotic proteins, such as NOXA and PUMA, thereby shifting leukemic cell survival reliance towards BCL-2 and ultimately sensitizing cells to VEN-mediated apoptosis [72].

Cyclin-dependent kinase (CDK) inhibition, and mostly CDK9 inhibition, leads to reduced transcription and, consequently, expression of MCL-1 [73]. Alvocidib, a multiple CDK inhibitor, has proven its efficacy in preclinical AML studies, while synergy with VEN has also been observed [74]. However, the combination of alvocidib with VEN in R/R AML (NCT03441555) has failed to provide a meaningful clinical benefit [75]. Dinaciclib, a more selective and potent CDK9 inhibitor, is currently being investigated in combination with VEN in R/R AML (NCT03484520). Other CDK inhibitors evaluated in clinical trials in combination with VEN include voruciclib (NCT03547115), fadraciclib (NCT05168904), AZD4573 (NCT03263637), RVU120 (NCT06191263/RIVER-81), and QHRD107 (NCT06532058).

Pevonedistat (PEV), a NEDD8-activating enzyme inhibitor, leads to enhanced NOXA expression and subsequent increased MCL-1 degradation [76]. The combination of PEV, AZA, and VEN has yielded an overall response rate (ORR) of 46.7% in R/R AML [77], while the addition of PEV to AZA and VEN in ND AML failed to show a clinical benefit [78]. Selinexor, an exportin 1 inhibitor that also leads to reduced MCL-1 levels, has been explored in AML patients in combination with HMAs and VEN [79]. Preliminary data from the use of triplet DEC/VEN/selinexor in ND and R/R AML patients have shown a complete response (CR) rate of 55.5% [80]. A phase 1b trial of VEN and selinexor in R/R AML has demonstrated modest responses with an ORR of 21% [81]. A trial of the second-generation eltanexor combined with VEN in R/R AML is underway (NCT06399640).

Combined inhibition of the BCL-2/MAPK pathway to induce MCL-1 degradation with MEK or MDM2 inhibitors represents another attractive option [82,83]. Unfortunately, a phase 1b trial of cobimetinib, a MEK1/2 inhibitor, combined with VEN in R/R AML patients has reported composite complete response (cCR) rates of 15.6% [84]. However, encouraging results have been reported in R/R AML with the combination of the MDM2 inhibitor idasanutlin with VEN, with cCR rates of 34.3% overall and 20% among patients with *TP53* mutations [85]. Trials with other MDM2 inhibitors, such as siremadlin and KRT-232, are currently being conducted (NCT03940352, NCT03041688).

Moreover, ^225^Ac-lintuzumab, which targets CD33, has shown activity in VEN-resistant cells through MCL-1 suppression [86], and is currently being investigated in combination with VEN (NCT03867682). Lastly, several promising novel agents have demonstrated significant efficacy in preclinical models but have not yet entered clinical trials; these agents include the anticancer quinolone (R)-WAC-224, which leads to caspase 3-mediated cleavage of MCL-1 [87,88], the sphingosine kinase 1 inhibitor MP-A08, which induces ceramide accumulation and integrated stress response (ISR) activation, ultimately leading to increased NOXA and NOXA-dependent MCL-1 degradation, and the antibiotic tedizolid, which, in combination with VEN, enhances ISR and leads to reduced OXPHOS [89,90,91].

Taken together, these studies indicate that indirect MCL-1 targeting can sensitize cells to VEN, but clinical efficacy remains variable and requires careful optimization of combinations.

#### 2.2.2. Metabolism-Targeting Strategies

Emerging therapeutic strategies aiming to alter the metabolism of leukemic cells may help overcome VEN resistance. As previously described, acquired VEN resistance is mediated by OXPHOS and sustained by FAO [30,31,32,33]. Preclinical studies have shown that combining OXPHOS inhibition enhances VEN activity [31,91].

Inhibition of STAT3, which regulates mitochondrial respiration, prompts reduced OXPHOS and is effective in overcoming resistance in preclinical models [91]. OPB-111077, a STAT3 inhibitor, has been investigated in combination with VEN and DEC in both ND and R/R AML, with preliminary results showing an ORR of 25% [92]. A phase 1 study exploring the safety and efficacy of danvatirsen, a selective antisense oligonucleotide inhibitor of STAT3 in combination with VEN in R/R AML, is ongoing (NCT05986240) [93]. Furthermore, the inhibition of Janus Kinase 2 (JAK2), which lies upstream of STAT3, with ruxolitinib combined with VEN with or without AZA in R/R AML patients has shown that this combination is safe, achieving a clinical benefit rate of 63% [94].

MicroRNA 126 (miR-126) maintains LSC function by promoting BCL-2-dependent FAO and OXPHOS, whereas its inhibition disrupts mitochondrial metabolism and triggers apoptosis [95]. Preclinical studies have shown synergy of miRisten, a CpG-conjugated anti-miR-126 oligonucleotide, with VEN, enabling the entry of miRisten into a clinical trial (NCT07025564) [95]. Pitavastatin, an inhibitor of 3-hydroxy-3-methylglutaryl coenzyme A (HMG-CoA), has been proven to be effective in counteracting VEN resistance, mostly by depleting geranylgeranyl pyrophosphate, ultimately resulting in apoptosis [96]. A phase 1 study of pitavastatin combined with VEN in AML patients (NCT04512105) has recently been completed and awaits results.

In addition, targeting NAD+ through inhibition of nicotinamide phosphoribosyltransferase (NAMPT), which is crucial for NAD+ biosynthesis, ultimately resulting in mitochondrial dysfunction, is another attractive option. KPT-9274, a NAMPT inhibitor, has shown promising efficacy and synergy with VEN in preclinical models [97]. A phase 1 study of KPT-9274 in R/R AML (NCT04914845) is currently ongoing. Finally, the combination of metformin, an antidiabetic biguanide that inhibits ETC I and OXPHOS, with VEN and cytarabine has displayed preliminary efficacy, particularly in patients with R/R AML [98].

Collectively, these findings suggest that targeting leukemic cell metabolism represents a promising strategy to overcome VEN resistance, with several approaches now entering early-phase clinical trials to evaluate safety and preliminary efficacy.

#### 2.2.3. Targeting Genetic and Epigenetic Mechanisms

Targeting epigenetic mechanisms with novel agents is also an appealing strategy to tackle VEN resistance. Oral decitabine/cedazuridine (DEC-C) has recently been approved for the treatment of myelodysplastic neoplasms [97]. Multiple clinical trials are evaluating DEC-C with VEN alone or in triplet combinations. Notably, frequent weekly lower-dose DEC combined with VEN achieved superior outcomes compared with standard HMA/VEN dosing, with activity in *TP53*-mutated disease, suggesting that sustained DNA methyltransferase 1 inhibition may help overcome VEN resistance [99,100].

Inhibition of lysine-specific demethylase 1 (LSD1), an enzyme implicated in methyl group removal from histone proteins, represents another emerging strategy [101]. Iadademstat, a novel LSD1 inhibitor, enhances blast differentiation and reduces LSC self-renewal by increasing histone H3 lysine 4 dimethylation (H3K4me2) levels, particularly in KMT2A-rearranged AML [102]. Data from preclinical studies have shown synergy with BCL-2i [102], and clinical trials are currently evaluating its use in combination with AZA and VEN in AML (NCT06357182, NCT06514261).

Additionally, chidamide, a selective histone deacetylase (HDAC) inhibitor that also induces MCL-1 downregulation, has shown promising efficacy when combined with AZA and VEN in overcoming resistance in AML [103]. Several clinical trials are currently investigating chidamide both in ND and R/R AML.

Tackling specific genetic alterations may also help circumvent VEN resistance. RAS inhibition with trametinib, an oral MEK inhibitor, has been shown to have preclinical synergy with VEN. However, a phase 2 trial of AZA/VEN/trametinib in heavily pretreated R/R AML has demonstrated only modest activity with a CR rate of 25% [104]. Finally, the combination of VEN with IDH inhibitors in patients with *IDH*-mutated (*IDH*^mut^) R/R AML has significantly improved outcomes, as will be discussed below.

Taken together, this data indicates that targeting genetic and epigenetic mechanisms can resensitize AML cells to VEN, with several early-phase clinical trials now exploring combinations in both genetically defined and high-risk patient groups.

**Table 1:** Therapeutic strategies used to overcome resistance to venetoclax in acute myeloid leukemia.

Strategy	Agent/Combination	Study Phase or Preclinical Model	Population	Clinical Trial Identifier *	Results	Ref.
**Strategies to overcome resistance to VEN**						
Direct MCL-1 inhibition						
Direct MCL-1 inhibitors	S64315 (MIK655) + AZA	1/2	ND AML ineligible for IC R/R AML	NCT04629443	No results posted	
	S64315 (MIK655) + VOB560 (novel BCLi)	1b	R/R AML	NCT04702425	CR in 2/29 patients	[68]
	Tapotoclax (AMG-176)	1	R/R AML	NCT02675452	Terminated	
	Tapotoclax (AMG-176) + VEN	1b	R/R AML	NCT03797261	Terminated due to increased cardiotoxicity	
	Murizatoclax (AMG-397)	1	R/R AML	NCT03465540	Terminated	
	AZD5991 +/− VEN	1/1b/2a, 3	R/R AML	NCT03218683	Terminated due to elevated laboratory troponin and low ORR	[71]
	PRT1419	1	R/R AML	NCT05107856	Terminated	
	PRT1419	1	R/R AML	NCT04543305	No results posted	
Indirect MCL-1 inhibition						
CDK inhibitors	Alvocidib + VEN	1b	R/R AML	NCT03441555	CR + CRi 11.4%	[75]
	Dinaciclib + VEN	1b	R/R AML	NCT03484520	Terminated	
	Voruciclib + VEN	1	R/R AML	NCT03547115	Unknown status	
	Fadraciclib + VEN	1/2	R/R AML	NCT05168904	Suspended	
	AZD4573	1	R/R AML	NCT03263637	Completed-no results posted	
	RVU120	2	R/R AML	NCT06191263	Recruiting	
	QHRD107 + AZA + VEN	2a	R/R AML	NCT06532058	Recruiting	
NEDD8-activating enzyme inhibitor	Pevonedistat + AZA + VEN	1	R/R AML	NCT04172844	ORR: 46.7%	[77]
	Pevonedistat + AZA + VEN	2	ND AML	NCT04266795	CR: 45% cCR: 77%	[78]
XPO1 inhibitor	Selinexor + VEN + DEC	NA **	ND AML R/R AML		CR: 55.5%	[81]
	Selinexor + VEN	1b	R/R AML	NCT03955783	ORR: 21%	[82]
	Eltanexor + VEN	1b	R/R AML	NCT06399640	Recruiting	
Combined MAPK/BCL-2 inhibition	Cobimetinib (MEK 1/2 inhibitor) + VEN	1b	R/R AML	NCT02670044	cCR:15.6%	[84]
	Idasanutlin (MDM2 inhibitor) + VEN	1b	R/R AML	NCT02670044	cCR: 34.3%	[85]
	KRT-232 (AMG-232) + VEN + DEC	1b	ND AML R/R AML	NCT03041688	Active—no results posted	
Anti-CD33	^225^Ac-lintuzumab + VEN	1/2	R/R AML	NCT03867682	Unknown status	
Metabolism targeting therapies						
STAT3 inhibitors	OPB-111077 + VEN + DEC	1b	ND AML R/R AML	NCT03197714	ORR: 25%	[92]
	Danvatirsen + VEN	1	R/R AML	NCT05986240	Recruiting	
	Ruxolitinib + VEN +/− AZA	1	R/R AML	NCT03874052	CBR: 63%	[94]
microRNAs	miRisten	1	R/R AML	NCT07025564	Recruiting	
HMG-CoA inhibitors	Pitavastatin + VEN	1	ND AML	NCT04512105	Completed-no results posted	
NAMPT inhibitors	KPT-9274	1	R/R AML	NCT04512105	Active—no results posted	
	Metformin + VEN + cytarabine	2	R/R AML ND AML	NCT06537843	cCR:83% in R/R AML cCR: 69% in ND AML	[98]
Epigenetic targeting						
DEC-C	DEC-C + VEN	1/2	AML not previously treated with HMA and/or VEN	NCT04657081	Active—no results posted	
	DEC-C + VEN	1/2	High risk AML	NCT04817241	Active—no results posted	
	DEC-C + VEN	2	R/R AML	NCT04975919	Active—no results posted	
	DEC-C + VEN	2	R/R AML ND AML	NCT04746235	Active—no results posted	
LSD1 inhibitor	Iadademstat + AZA + VEN	1	ND AML	NCT06514261	Recruiting	
HDAC	Chidamide + AZA + VEN	ΝA **	ND AML	NCT06386302	Recruiting	
	Chidamide + AZA + VEN	2	R/R AML	NCT05305859	Recruiting	
RAS inhibition	Trametinib + AZA + VEN	2	R/R AML	NCT04487106	CR: 25%	[104]
Novel BCL-2 inhibitors	Navitoclax + VEN + DEC	1	R/R AML	NCT05222984	CRh/CRi: 20%	
	Lisaftoclax (APG-2575) + AZA	1b/2	R/R AML	NCT04964518	ORR: 29.2% in VEN refractory AML	[64]

* clinicaltrials.gov. ** Multicenter Randomized. VEN, venetoclax; MCL-1, myeloid cell leukemia-1; AZA, azacitidine; ND, newly diagnosed; AML, acute myeloid leukemia; IC, intensive chemotherapy; R/R, relapsed/refractory; BCLi, BCL-2 INHIBITOR; CR, complete response; ORR, overall response rate; CDK, cyclin-dependent kinase; CRi, complete response with incomplete hematologic recovery; NEDD8, neural precursor cell expressed developmentally downregulated protein 8; cCR, composite complete response; XPO1, exportin 1; DEC, decitabine; BCL-2, B-cell lymphoma 2; MDM2, mouse double minute 2; STAT3, signal transducer and activator of transcription 3; CBR, complete best response; HMG-CoA, 3-hydroxy-3-methylglutaryl coenzyme A; NAMPT, nicotinamide phosphoribosyltransferase; HMA, hypomethylating agents; DEC-C, oral cedazuridine/decitabine; LSD1, lysine-specific histone demethylase 1A; HDAC, histone deacetylase; CRh, complete response with partial hematologic recovery; NA, not applicable.

## 3. FLT3 Inhibitors

FLT3 is a receptor tyrosine kinase involved in the regulation of cell proliferation, differentiation, and survival through the activation of intracellular signaling pathways upon the binding of the FLT3 ligand (FL) [105]. Activating mutations of *FLT3* are among the most frequent genetic abnormalities in AML, occurring in up to 30% of patients [106]. These can be either internal tandem duplications (*FLT3*-ITD) in the juxtamembrane domain or point mutations in the tyrosine kinase domain (*FLT3*-TKD) [106,107,108]; their presence has long been recognized to confer poor prognosis [109].

Targeting FLT3 has become an important component of AML treatment, and several first- and second-generation FLT3 inhibitors (FLT3i) have been developed [110,111]. Based on their mechanism of action, FLT3i can be further divided into type 1 inhibitors which enter into the ATP-binding site following receptor activation and type 2 inhibitors that interact with the receptor in its inactive conformation [110]. Subsequently, type 1 inhibitors, such as midostaurin, gilteritinib, crenolanib, and lestaurtinib, are active against both *FLT3*-ITD and *FLT3*-TKD mutations, whereas type 2 inhibitors, such as quizartinib and sorafenib, are only active against *FLT3*-ITD mutations [110]. Although FLT3i have significantly improved outcomes in *FLT3*^mut^ AML, a substantial proportion of patients fail to respond or relapse [112,113,114,115,116,117], indicating that resistance, both intrinsic and acquired, remains a major challenge in these patients.

### 3.1. Mechanisms Underlying Resistance to FLT3i

#### 3.1.1. Bone Marrow Microenvironment-Mediated Resistance

The bone marrow (BM) stromal microenvironment plays a pivotal role in promoting resistance to FLT3i. Elevated FL levels, particularly observed in relapsed AML patients, represent a key driver of primary resistance by preserving persistent activation of the FLT3/MAPK pathway, thus sustaining leukemic cell survival [118]. A preclinical model has demonstrated that exogenous FL diminishes the efficacy of FLT3i, including lestaurtinib, midostaurin, and sorafenib [14]. Moreover, high FL levels have been associated with increased activation of STAT1, STAT3, and STAT5 signaling pathways, thereby promoting survival and contributing to resistance [119,120]. Collectively, these findings highlight FL-driven signaling as a potent mechanism of resistance.

Several other microenvironment-derived factors have been linked to the development of resistance to FLT3i. Activation of the fibroblast growth factor 2 (FGF2)/FGF receptor 1 (FGFR1) axis has been shown to stimulate sustained MAPK signaling in *FLT3*-ITD AML cells [121]. Other studies have reported an increased FGF2 expression at relapse of *FLT3*^mut^ AML [121]. Importantly, a direct link has been demonstrated between the activation of the FGF2/FGFR1 axis, high levels of FL, and resistance to quizartinib [122,123]. Taken together, these findings suggest that both FGF2 and FLT3 signaling converge on the MAPK/AKT pathway, whose constitutive activation might be the final common node of resistance. Lastly, another study in *FLT3*-ITD AML cells has demonstrated that a pro-inflammatory microenvironment confers resistance to FLT3i and is primarily mediated by interferon γ (IFN-γ), which activates STAT1, ultimately leading to upregulation of anexelekto (AXL), a member of receptor tyrosine kinases (RTKs), and enhances cell survival despite FLT3 inhibition [124].

Upregulation of chemokines, such as CXC motif chemokine ligand 12 (CXCL12), as well as the elevated expression of its receptor CXCR4 on leukemic cells, has also been shown to promote proliferation, thereby contributing to resistance [125]. Moreover, blasts with higher levels of CXCR4 have reduced sensitivity to FLT3i [126]. The interference between FL and CXCL12 leads to abnormal phosphorylation of the p42/p44/MAPK/AKT pathway [127]. Finally, cytochrome P450 3A4 (CYP3A4), an enzyme highly expressed in BM mesenchymal stromal cells, has also been recognized as a mediator of resistance to FLT3i. In detail, stromal CYP3A4 activity has been associated with resistance to sorafenib, quizartinib, and gilteritinib in *FLT3*^mut^ AML, whereas use of clarithromycin, a strong CYP3A4 inhibitor, has restored sensitivity [128].

#### 3.1.2. Transcriptional Heterogeneity and Transcription Factor-Driven Mechanisms of Resistance

Recent studies have highlighted that pre-existing intrinsic transcriptional heterogeneity might be an important contributing factor to the early occurrence of resistance [129]. Single-cell transcriptional profiling has revealed the presence of pre-existing resistant subpopulations characterized by distinct gene expression profiles associated with resistance to FLT3i, including upregulation of G1 to S phase transition 1 (GSPT1) [129]. Notably, the targeting of GSPT1 resensitized cells to FLT3i and also exhibited strong synergy with FLT3i both in vitro and in xenograft *FLT3*^mut^ AML models [129]. Finally, the transcription factor (TF) OCT4 has been shown to enhance abnormal FLT3 signaling through transcriptional activation of TF NANOG, thus leading to resistance [130]. Importantly, FLT3 inhibition has resulted in upregulation of OCT4 and NANOG in *FLT3*^mut^ AML cells, thus suggesting that these TFs might not only be responsible for intrinsic resistance to FLT3i but also serve as adaptive mechanisms to overcome FLT3i [130].

#### 3.1.3. FLT3 Mutations

In detail, these mutations are more commonly found within the TKD, particularly at residues D835 and Y842 of the activation loop (A-loop), thus maintaining the TK domain in its active form and ultimately impeding binding of type 2 inhibitors, since they bind FLT3 only in its inactive conformation [131,132,133]. Secondary D835 mutations, such as D835Y, D835V, and D835F, are found in up to one-third of AML patients with acquired resistance to quizartinib and sorafenib, and have been correlated with inferior OS [131,132,133]. Of note, multiple coexisting TKD mutations have been reported, thus indicating polyclonal resistance [131].

Other less frequently reported mutations include the TKD N676K and N841K mutations as well as the extracellular domain mutation K429E, which have been associated with resistance to specific inhibitors, such as midostaurin and crenolanib, respectively [134,135]. Notably, crenolanib appears less likely to induce A-loop mutations and retains activity against many D835 variants, thereby rendering it a potent inhibitor in these cases [134,135]. Finally, the F691L gatekeeper mutation has been associated with universal resistance to nearly all FLT3i [136,137]. Therefore, identifying these mutation patterns in patients treated with FLT3i at relapse is very important, since switching between inhibitor types may help circumvent resistance.

#### 3.1.4. Activation of Alternative Signaling Pathways Bypassing FLT3 Inhibition and Other Mechanisms

Beyond on-target resistance, secondary off-target resistance can also arise, and is frequently driven by acquired activating mutations in downstream signaling pathways that bypass dependence on FLT3 signaling. Mutations in the RAS/MAPK pathway genes, including NRAS, KRAS, and CBL, have been reported in patients with resistance to gilteritinib [138,139]. Furthermore, mutations in epigenetic regulators, such as *IDH1*, *IDH2*, and ten-eleven translocation 2 (*TET2*), have also been reported in association with resistance to gilteritinib and crenolanib [135,136]. Importantly, the loss of original FLT3 mutations along with the acquisition of new ones has been documented in some patients at relapse, suggesting that resistance may emerge through clonal switching [138].

Resistance to FLT3i has also been associated with upregulation of the JAK/STAT pathway. Specifically, JAK mutations such as V617F, S703I, R724H, and A573V have been identified in 46% of patients who developed resistance to midostaurin and quizartinib [140]. In addition, suppression of Src homology region 2 domain-containing phosphatases (SHP1) and SHP2, which are negative regulators of the JAK pathway, has been observed in cases of *FLT3*-TKD [141]. Furthermore, epigenetic silencing of suppressors of cytokine signaling (SOCS) 1 and SOCS2 has been reported [141]. Collectively, these alterations lead to amplification of STAT5-driven survival signaling and contribute to resistance [140,141]. Moreover, growth arrest-specific factor 6 (GAS6)-mediated upregulation of AXL induces activation of the PI3K/AKT, RAS/MAPK, and STAT pathways and has been correlated with resistance to midostaurin and quizartinib [142]. Upregulation of the proto-oncogene serine/threonine protein kinase family PIM, whose expression is strongly induced by *FLT3*-ITD, leads to increased MCL-1 expression and has also been linked to resistance. [143]. Additionally, FLT3i induces histone deacetylase 8 (HDAC8) upregulation through activation of FOXO1 and FOXO3, which subsequently leads to p53 inactivation, sustaining survival despite FLT3 inhibition [144]. Lastly, other secondary off-target resistance mechanisms include spleen tyrosine kinase (SYK) activation, enhanced drug efflux via P-glycoprotein, and upregulation of tescalcin type 1 Na^+^/H^+^ exchange channel (TESC) protein [145,146,147].

### 3.2. Strategies to Overcome FLT3 Inhibitor Resistance

#### 3.2.1. Switching FLT3 Inhibitor Type

Switching between type 1 and type 2 FLT3i may help overcome resistance, given that cells resistant to type 2 inhibitors due to TKD mutations may retain sensitivity to type 1 inhibitors. Other strategies aiming to tackle the resistance to FLT3i include the use of novel FLT3i and the employment of combination treatments targeting parallel or downstream signaling pathways or other adaptive resistance mechanisms. Several agents have been explored in preclinical settings; only a few have proceeded in clinical trials. Table 2 summarizes important investigational agents used to tackle resistance to FLT3i.

#### 3.2.2. Novel FLT3i

In recent years, several novel FLT3i have been developed with demonstrated efficacy, mostly in preclinical models. Sitravatinib is a novel FLT3i with both in vitro and in vivo efficacy in *FLT3*-ITD AML, even in the presence of cytokines, by inhibiting ERK and AKT more potently [148]. Sitravatinib has also displayed synergy with VEN in preclinical models by reducing MCL-1 expression and inhibiting MAPK and AKT pathways [149]. Remarkably, sitravatinib has also exhibited a potent inhibitory effect on the F691L gatekeeper mutation, which confers resistance to all other FLT3i [148].

Activity against F691L has also been demonstrated with ningetinib, another novel FLT3i, with efficacy against TKD mutations, such as D835Y/V and Y842C, by inhibiting FLT3 and its downstream pathways, including AKT, ERK, and STAT5 [150]. FF-10101 is an FLT3i that irreversibly binds to C695 residue and has exhibited activity against F691L and D835 mutations [151]. Importantly, FF-10101 has been shown to circumvent BM microenvironment-associated resistance by reducing the release of growth factors in the niche [151]. A phase 1 study of FF-10101 in R/R AML has demonstrated cCR rates of 10% and ORRs of 12.5% with responses in patients progressing on other FLT3i [152]. Other recently developed FLT3i with proven efficacy in preclinical models include CCM-405, CCM-445, and LT-171-861 [153,154].

#### 3.2.3. Combined Approaches: Targeting Signaling Pathways and Bone Marrow Microenvironment

Combining FLT3i with agents inhibiting compensatory signaling pathways, such as MEK-ERK and PI3K/AKT, represents another promising approach. Combined targeting of FLT3 and AXL, whose upregulation has been shown to confer resistance, offers the possibility to improve treatment outcomes [142]. Among AXL inhibitors, TP-0903, CTS2016, DAXL-88, and DAXL-88-MMAE have displayed both in vivo and in vitro activity in *FLT3*^mut^ AML resistant to FLT3i and have also exerted synergy with FLT3i. Synergistic effects of CTS2016 combined with AZA and VEN have also been documented. Tamnorzatinib (ONO-7475), a dual AXL/Mer inhibitor, has also displayed synergy with VEN in *FLT3*-ITD AML in preclinical models [155,156,157]. However, a phase 1/2 clinical trial (NCT03176277) evaluating tamnorzatinib in R/R AML, including *FLT3*^mut^ patients, as both monotherapy or combined with VEN, failed to show benefit and was prematurely terminated [158].

Dual JAK2 and FLT3 inhibition also represents an attractive option in resistant cases, since evidence suggests that JAK2 inhibitors can mitigate FLT3i resistance [159]. Pacritinib, a dual JAK2/FLT3 inhibitor, can overcome resistance, based on in vitro and in vivo studies, and has also demonstrated preliminary activity against *FLT3*-ITD AML in a phase 1 study in combination with chemotherapy or decitabine [160]. Similar results have been documented with the use of other inhibitors in preclinical models, such as momelotinib and compound 11r, a dual JAK2/FLT3 inhibitor [159,161].

Concomitant inhibition of MEK-ERK1/2 and FLT3 has also been explored. Trametinib has shown potency in overcoming resistance to FLT3i, while also displaying synergy with midostaurin [162]. Targeting of aberrant RAS/MAPK signaling has also been investigated. In particular, the RAS(ON) multi-selective inhibitor daraxonrasib (RMC-7977) has displayed significant efficacy in AML cell lines and has resensitized cells to gilteritinib, while inhibiting the outgrowth of gilteritinib-resistant RAS-mutant clones [163].

Targeting the CXCL12/CXCR4 axis represents another option. Investigational CXCR4 antagonists, such as LY2510924 and GMI-1359, have exhibited potency in circumventing resistance to FLT3i, which is mostly mediated by disrupting the BM microenvironment [164,165]. Dual inhibition of MDM2 and *FLT3*-ITD with milademetan and quizartinib showed efficacy in preclinical studies and in a phase 1 study, with reported CR with incomplete hematologic recovery rates of 40% [166].

Finally, in vitro studies of CDK4/6 inhibitors, such as palbociclib and abemaciclib, have demonstrated efficacy in overcoming resistance to FLT3i [167,168]. A clinical trial of palbociclib with sorafenib, decitabine, or dexamethasone is currently ongoing (NCT03132454), whereas a phase 1/2 trial of palbociclib and CPX-351 in patients with AML has reported CR rates of 62.5% in R/R AML patients, including patients with *FLT3* mutations [169].

Collectively, these studies highlight that combination strategies targeting FLT3 along with compensatory signaling pathways hold promise to overcome resistance, though translation to clinical benefit has been variable, underscoring the need for careful selection of combinations and patient populations in ongoing and future clinical trials.

**Table 2:** Therapeutic strategies used to overcome resistance to FLT3 inhibitors in acute myeloid leukemia.

Strategy	Agent/Combination	Study Phase or Preclinical Model	Population	Clinical Trial Identifier *	Results	Ref.
**Strategies to overcome resistance to FLT3i**						
FLT3 inhibition						
Novel FLT3i	Ningetinib	1b	R/R *FLT3^mut^* AML	NCT03125876	Terminated	
	FF-10101	1	R/R AML with *FLT3*^mut^ patients	NCT03194685	cCR: 10% ORR: 12.5%	[152]
Combined FLT3 and signaling pathways inhibition						
AXL inhibitors	Tamnorzatinib (ONO-7475) +/− VEN	1/2	R/R AML with *FLT3*^mut^ patients	NCT03176277	CRi: 7.1% in VEN-resistant cohort MLS: 7.1% in VEN-resistant cohort	[158]
JAK2 inhibitors	Pacritinib + cytarabine and/or daunorubicin DEC	1	R/R AML with *FLT3*^mut^ patients	NCT02323607	CR: 40% in cohort A CR: 0% in cohort B	[160]
MEK-ERK inhibitors	Trametinib	CL and PS	*FLT3*^mut^ AML	ΝA	Synergistic effect with midostaurin	[162]
	Daraxonrasib (RMC-7977)	CL	AML	ΝA	Restoration of sensitivity to FLT3i	[163]
CXCR4 inhibitors	GMI-1359	PDX	*FLT3*^mut^ AML	ΝA	Enhanced antileukemia effect of sorafenib	[164]
MDM2 inhibitors	Milademetan + Quizartinib	1	ND and R/R *FLT3*-ITD AML	NCT03552029	CRi: 40%	[166]
CDK4/6 inhibitors	Palbociclib + Sorafenib, DEC or dexamethasone	1	R/R AML	NCT03132454	Active—no results posted	
	Palbociclib + CPX-351	1/2	ND and R/R AML	NCT03844997	CR: 62.5% in R/R AML, including FLT3^mut^ patients	[169]
Multi-kinase inhibitors	Pexidartinib	1/2	R/R *FLT3*-ITD AML	NCT01349049	cCR: 11%	[170]
	Foretinib	Mouse model and PDX	*FLT3*-ITD AML		Strong efficacy including potent activity against secondary mutations of FLT3-ITD	[171]
	Tuspetinib +/− VEN	1/2	R/R AML	NCT03850574	ORR: 23.1% in FLT3-mutated patients with prior FLT3i treatment	[172]
	Luxpetinib	CL, PS	AML		Greater potency than other FLT3i, including cases with FLT3 mutations	[173]
	CCT241736	PDX and PS	*FLT3*-ITD and *FLT3*-TKD AML		Inhibition of tumor growth, including quizartinib-resistant cases	[174]
	Tirbanibulin (KX2-391)	Mouse model and PS	*FLT3*-ITD and *FLT3*-TKD AML		Potent inhibitory effects in FLT3^mut^ cells with resistant mutations	[175]
PIM inhibitors	AZD1208	1a/1b	R/R AML	NCT01489722	Terminated due to lack of efficacy	[176]
	SGI-1776	1	R/R AML	NCT01239108	Terminated due to safety issues	
Epigenetic targeting						
HDAC inhibitors	IHCH9033, compound 22, compound 25h, decursin)	CL and PDX	*FLT3*^mut^ AML		Synergistic effects when combined with FLT3i	[144,177,178,179]
Immunotherapeutic approaches						
Anti-FLT3 CAR-T cells	TAA05 Cell Injection	1	R/R AML	NCT05445011	Recruiting	
	CI-135 CAR-T Cell	1	R/R AML	NCT06760260	Recruiting	
	HG-CT-1	1	R/R AML	NCT06786533	Recruiting	
anti-FLT3 × anti-CD3 BiTEs	CLN-049	1	R/R AML	NCT05143996	ORR: 57%	
	AMG 427	1	R/R AML	NCT03541369	Terminated	

* clinicaltrials.gov. VEN, venetoclax; newly diagnosed; AML, acute myeloid leukemia; R/R, relapsed/refractory; CR, complete response; ORR, overall response rate; CDK, cyclin-dependent kinase; CRi, complete response with incomplete hematologic recovery; cCR, composite complete response; DEC, decitabine; MDM2, mouse double minute 2; *FLT3*, FMS-like tyrosine kinase 3; FLT3i, FLT3-inhibitor; FLT3^mut^, FLT3-mutated; AXL, anexelekto; MLS, morphologic leukemia-free state; JAK2, Janus kinase 2; CL, cell lines; PS, patient samples; CXCR4, C-X-C chemokine receptor type 4; PDX, patient-derived xenograft model; FLT3-ITD, FLT3-internal tandem duplication; FLT3-TKD, FLT3-tyrosine kinase domain; CAR-T cells, chimeric antigen receptor-T cells; BiTE, bispecific T-cell engager.

#### 3.2.4. Multi-Kinase and PIM Inhibitors

Several multi-kinase inhibitors have been investigated to overcome resistance to FLT3 inhibition. Pexidartinib, a type 1 FLT3i also targeting KIT and colony-stimulating factor-1 receptor, has displayed potent clinical activity and safety in a phase 1/2 clinical trial of *FLT3*-ITD-mutant R/R AML patients, including those with F691 gatekeeper mutations [170]. However, its efficacy was particularly influenced by mutations in the activation loop. Foretinib is another multi-kinase inhibitor with promising efficacy in AML cell lines and has demonstrated activity against secondary mutations that confer resistance to quizartinib and gilteritinib [171]. Importantly, a phase 1/2 trial of tuspetinib, an oral multi-kinase inhibitor, in R/R AML patients has shown ORRs of 23.1% in *FLT3*^mut^ patients previously treated with FLT3i [172]. Other investigational multi-kinase inhibitors include luxeptinib, CCT241736, and KX2-39 [173,174,175]. Luxpetinib is a highly potent inhibitor of all FLT3 forms, while it also targets additional kinases implicated in survival and resistance pathways [173]. CCT241736, a potent dual inhibitor of FLT3 and Aurora kinases, has exhibited efficacy in samples from AML patients, including those resistant to quizartinib [174]. Finally, KX2-391 (tirbanibulin) is an oral non-ATP-competitive inhibitor of Src kinase and tubulin polymerization displaying preliminary activity against resistant *FLT3*-ITD AML [175].

The employment of PIM-inhibitors has also been explored but has been met with unsatisfying results. AZD1208, a pan-PIM inhibitor, has demonstrated efficacy combined with quizartinib in *FLT3*-ITD AML cells [176]. However, preliminary results of a phase 1 study have indicated no benefit in AML [176]. Furthermore, a trial of another pan-PIM inhibitor, SGI-1776, has been withdrawn due to safety issues regarding QT prolongation (NCT01239108).

#### 3.2.5. Epigenetic Targeting

HDAC inhibitors have been shown to effectively overcome resistance to FLT3i in AML cells while also exerting synergistic effects with FLT3i [144]. IHCH9033 is a novel selective class 1 HDAC inhibitor that suppresses DNA repair pathways in *FLT3*^mut^ AML cells while also stimulating the acetylation of HSP90, thereby resulting in FLT3 ubiquitination and proteasomal degradation [177]. IHCH9033 has proved its efficacy in FLT3i-resistant cells and displayed synergistic activity with quizartinib [177]. Similar results have been reported in preclinical studies of compound 22, a selective HDAC8 inhibitor, and compound 25 h, a dual FLT3 and HDAC inhibitor [144,178]. Decurcin, a pyranocoumarin, also leads to proteasome-mediated FLT3-ITD degradation by increasing the expression of ubiquitin conjugase UBE2L6 [179].

#### 3.2.6. Immunotherapy Approaches and Other Investigational Agents

Lastly, several other investigational agents are currently being explored in preclinical studies in resistant *FLT3*^mut^ AML, including ceramide transfer protein (CERT) inhibitors, the antipsychotic chlorpromazine, sphingosine-1-phosphate receptor modulators, heme oxygenase 1 inhibitors, and NLRP3 inhibitors [180,181,182,183,184]. Furthermore, immunotherapeutic agents have also been investigated, including FLT3 chimeric antigen receptor (CAR)-T cells and FLT3-directed bispecific T-cell engager (BiTE), both of which have displayed efficacy in preclinical AML models [185,186]. Several ongoing studies are evaluating the efficacy of FLT3 chimeric antigen receptor (CAR)-T cells in R/R AML (NCT05445011, NCT06760260, NCT06786533, NCT05023707). CLN-049, a novel anti-FLT3 × anti-CD3 BiTE, has displayed ORRs of 57% in a phase 1 study in heavily pretreated R/R AML patients, thereby leading to fast-track designation by the FDA [187,188]. A study of another anti-FLT3 x anti-CD3 BiTE, AMG 427 (NCT03541369), has been prematurely suspended. Nevertheless, immunotherapeutic approaches are promising in R/R *FLT3*^mut^ AML, even though their clinical application remains in its early stages. These preclinical and clinical studies suggest that novel immunotherapeutic approaches offer promising strategies to overcome resistance in *FLT3*^mut^ AML, though clinical validation remains in early stages.

## 4. IDH Inhibitors

IDH1 and IDH2 are metabolic isoenzymes that play a central role in cellular metabolism [189]. Under physiologic conditions, both enzymes catalyze the oxidative decarboxylation of isocitrate to a-ketoglutarate (α-KG) and reduce NADP^+^ to NADPH in the TCA cycle [190]. Furthermore, α-KG possesses antioxidant properties, and the produced NADPH helps maintain cellular redox balance by reducing glutathione through glutathione reductase [189]. Moreover, α-KG serves as a cofactor for a wide range of α-KG-dependent dioxygenases, including TET, JmjC domain-containing histone demethylases (JmjC KDMs), and propyl hydroxylases (PHD), which are involved in epigenetic modifications such as DNA and histone demethylation, as well as hypoxia sensing [189,190,191].

*IDH1/2* mutations have been identified both in solid tumors and hematologic malignancies, including AML [192,193,194]. They can be found in 8–14% and 9–19% of AML cases, respectively. R132H is the most frequent *IDH1* variant, whereas R140Q and R172K constitute the majority of *IDH2* mutations, which may arise at different stages of leukemogenesis. [192,193,194].

Importantly, *IDH* mutations result in neomorphic enzymatic activity and lead to the conversion of a-KG into the oncometabolite 2-hydroxyglutarate (2-HG), which inhibits a-KG-dependent enzymes (e.g., TET and JmjC KDM), leading to DNA and histone hypermethylation, impaired cellular differentiation and disruption of alkylated DNA repair [195,196,197]. However, although these mutations promote oncogenic pathways, they are not sufficient to initiate leukemogenesis and usually require additional driver mutations [198,199]. Growing understanding of the contribution of *IDH1/2* mutations to AML pathogenesis has led to the development of IDH inhibitors (IDHi). These agents induce stabilization of the open conformation of mutant IDH, leading to the loss of its catalytic ability and subsequently prompting differentiation of blast cells [200,201]. Moreover, these drugs result in a significant reduction of 2-HG [200,201].

Ivosidenib, an IDH1 inhibitor (IDH1i), is approved for the treatment of both ND and R/R IDH1^mut^ AML [202,203]. Olutasidenib, another IDH1i, has recently been approved by the FDA for the treatment of R/R IDH1^mut^ AML [204]. Similarly, enasidenib, an IDH2 inhibitor (IDH2i), is approved for the treatment of R/R IDH2^mut^ AML [205]. Notably, the combination of ivosidenib with AZA has also yielded excellent clinical outcomes in ND IDH1-mutated AML, with reported CR and ORRs of 57% and 76%, respectively [206]. Nevertheless, some AML patients either fail to respond to IDHi or eventually relapse.

### 4.1. Mechanisms of Resistance to IDHi

Resistance to IDHi can be either intrinsic or acquired and represents a major therapeutic barrier. Importantly, multiple resistance mechanisms may occur concurrently, highlighting the complex nature of leukemic responses to IDH inhibition.

#### 4.1.1. Mutations in RTK Signaling Pathways

Mutations in RTK pathway genes have been identified as a key contributor to primary resistance to ivosidenib. The co-occurrence of *IDH1* mutations with mutations in RTK pathway genes, such as *NRAS*, *KRAS*, *PTPN11*, and *FLT3*, has been correlated with a lower probability of achieving a response following ivosidenib treatment [207,208]. Importantly, RTK pathway mutations also emerge at relapse in 35% of patients who have been previously treated with ivosidenib and achieved CR, thus suggesting that these mutations also confer acquired resistance to ivosidenib [208]. Likewise, mutations in *NRAS*, as well as in genes implicated in the MAPK pathway, have also been associated with enasidenib failure [209]. The biological mechanisms through which these mutations drive resistance remain incompletely understood; however, several explanations have been proposed. Activation of the RTK pathway may induce proliferation and survival signals that are strong enough to bypass cellular dependence on 2-HG. Secondly, these mutations may help sustain a differentiation block persisting even after treatment initiation with IDHi. Lastly, *IDH1/2* mutations may activate components of RTK signaling, which may not be reversed by IDHi in the presence of concurrent RTK pathway mutations [209].

#### 4.1.2. Mutations in WT1 Driving Resistance Though TET2 Impairment

A recent study of *IDH*^mut^ AML in a patient-derived xenograft model treated with IDHi has demonstrated a significant association between Wilms’ Tumor 1 (*WT1*) gene mutations and resistance to ivosidenib [210]. *WT1* mutations result in impaired TET2 function, rendering leukemic cells independent of 2-HG-mediated TET2 suppression to sustain their undifferentiated state [209,210]. However, the clinical relevance of *WT1* mutations remains unclear. In a study of patients with *IDH1*^mut^ R/R AML treated with ivosidenib, a non-statistically significant trend toward lower composite CR rates was observed in patients with *WT1* mutations compared with *WT1*-wild-type patients [210].

#### 4.1.3. Leukemia Stemness

Leukemia stemness represents another important mechanism of intrinsic resistance to IDHi. A multipronged genomic analysis of specimens from *IDH*^mut^ AML patients has shown that gene promoters related to transcriptional regulation of leukemia stemness display a hypermethylation phenotype driven by the forkhead box protein C1 (FOXC1), CD99, and DNMT3A, as well as by co-occurring mutations of transcription factors regulating hematopoietic differentiation, such as RUNX1, CEBPA, and GATA2 [211]. Importantly, this increased stemness has been associated with intrinsic IDHi resistance [210]. Finally, *IDH* mutations promote a stem cell-like phenotype by disrupting the Wnt/β-catenin signaling pathway in a 2-HG-dependent manner, thereby preventing cell differentiation and maintaining LSCs [212,213].

#### 4.1.4. Secondary Mutations and Additional Cytogenetic Lesions in Acquired Resistance

Besides the mutations in RTK pathway genes, secondary resistance may arise due to other acquired mutations in several genes, including *U2AF1*, *RUNX1*, *GATA2*, *BCL11A*, *BCORL1*, *NFKB1*, *DDX1*, *DHX15*, *MTUS1*, and *DEAF1* [214]. Increased variant allele frequency of *CSF3R*, *FLT3*, and *CBL* has also been shown to confer acquired resistance [214]. Moreover, monosomy 7 or partial deletion of chromosome 7 has been linked to increased relapse after enasidenib treatment [214].

#### 4.1.5. Isoform Switching

Isoform switching, either from mutant IDH1 to mutant IDH2 or vice versa, has been identified as another important mechanism of acquired resistance in both solid tumors and HMs [215]; however, its exact frequency remains unclear. Cells may acquire mutations in the alternate IDH isoform, bypassing IDH inhibition and restoring 2-HG production [215]. A case series has reported two patients with R/R AML with *IDH1*-R132C mutation who initially responded to ivosidenib but relapsed with the emergence of neomorphic mutation *IDH2*-R140Q, along with increased 2-HG levels [215]. Conversely, a patient with the *IDH2*-R132C mutation achieved remission with enasidenib but subsequently relapsed after acquiring an *IDH1*-R132C mutation [215]. Importantly, he achieved a transient response with a dual IDH inhibitor, thus suggesting that dual IDH inhibition may represent a promising combination.

#### 4.1.6. Second-Site Dimer Interface IDH Mutations That Weaken Drug Binding

Acquired resistance to IDH2i may also occur due to second-site mutations at the dimer interface [34]. In a previous report, resistance to enasidenib was associated with the emergence of second-site *IDH2* mutations Q316E and I319M that disrupt inhibitor binding and restore 2-HG. Interestingly, these resistance mutations occurred in trans, with the resistance-conferring variants arising in the *IDH2* allele lacking the neomorphic R140Q mutation [216]. This data has also been validated in molecular dynamics studies showing that, when occurring in trans, these mutations confer resistance by weakening a multitude of drug–protein interactions [217]. Secondary *IDH1* mutations, such as S280F, that alter inhibitor binding at the IDH1 dimer interface and lead to higher 2-HG production, have also been reported in association with resistance to ivosidenib [218]. Intriguingly, certain *IDH1* mutations, such as R132, display poor response to standard IDH1i due to unique structural features, including dihedral angle changes in the dimer interface and buried surface area charges that reduce inhibitor affinity [219].

#### 4.1.7. Metabolic Rewiring as a Mechanism of Secondary Resistance

Finally, secondary resistance to IDHi may stem from metabolic adaptations. *IDH*^mut^ AML cells display enhanced mitochondrial metabolism, including an increase in ETC I activity and TCA cycle intermediates [220]. Despite IDH inhibition and reduction of 2-HG production, these cells sustain high OXPHOS and FAO, which is primarily mediated by the inhibition of Akt and the increased activity of peroxisome proliferator-activated receptor-γ coactivator-1 (PGC1a), thereby maintaining energy production and survival [220]. Notably, the combination of OXPHOS inhibitors with IDHi has improved outcomes in vivo [220]. Moreover, CD44 is upregulated in *IDH*^mut^ AML cells and induces metabolic rewiring by activating the pentose phosphate pathway, while also inhibiting glycolysis, to ensure efficient NADPH generation for 2-HG production [221]. These metabolic alterations may also promote resistance to IDHi [221].

### 4.2. Strategies to Overcome Resistance to IDHi

Considering the multifaceted mechanisms underlying IDHi resistance, therapeutic strategies increasingly focus on combination approaches that simultaneously address resistance pathways. Key strategies are summarized in Table 3.

#### 4.2.1. Enhancing IDH Inhibition with Hypomethylating Agents and BCL-2 Blockade: Doublet and Triplet Therapy Approaches

Combining HMAs with IDHi in *IDH*^mut^ AML has become an area of active clinical investigation, with studies reporting a significant improvement in clinical outcomes [222,223]. Importantly, long-term results from the phase 3 AGILE study, which evaluated the addition of ivosidenib to AZA in ND *IDH1*^mut^ AML, have confirmed the combination’s efficacy, reporting a significantly improved OS in the combination arm compared to the AZA-placebo arm [222]. Correspondingly, the combination of AZA and enasidenib has displayed encouraging results in patients with ND *IDH2*^mut^ AML, with reported cCR rates of 66% [223]. Another phase 2 study of AZA and enasidenib in patients with both ND and R/R *IDH2*^mut^ AML has recorded a cCR rate of 100% and 58%, respectively. Notably, the combination of AZA and ivosidenib was shown to induce responses in patients harboring *NRAS*, *KRAS*, and *PTPN11* co-mutations, which have been associated with IDH1i resistance [208].

These observations are supported by a biological rationale, explaining the synergistic activity of HMAs and IDHi. HMAs act cooperatively with IDHi by inducing DNA hypomethylation and re-expression of silenced tumor suppressor genes [224]. Preclinical studies have demonstrated that this combination enhances leukemic cell differentiation and depletion [224]. Furthermore, IDH inhibition promotes cycling of LSCs and upregulation of the pyrimidine salvage pathway, thereby leading to increased sensitivity to AZA [225]. Finally, the coadministration of AZA with IDH1i inhibits the MAPK/ERK and RB/E2F pathways [226]. Collectively, the concurrent use of HMAs and IDHi addresses several aspects of IDHi resistance, such as the hypermethylated phenotype and activation of other signaling pathways, making it a particularly promising approach.

As previously mentioned, *IDH*^mut^ AML increases dependence on BCL-2, thereby augmenting sensitivity to VEN [55]. A phase 1b/2 study evaluating the combination of VEN and enasidenib in *IDH2*^mut^ AML patients reported a CR and ORR of 57% and 70%, respectively [227]. Furthermore, administration of VEN with HMAs has yielded responses in *IDH*^mut^ AML patients [55]. Hence, the use of triplet therapy with HMA, VEN, and IDHi represents another potent combination strategy. Several ongoing trials are currently evaluating the safety and efficacy of doublet or triple regimens in both ND and R/R *IDH*^mut^ AML (NCT06611839, NCT05907057, NCT05756777, NCT07075016, NCT03471260, NCT04774393, NCT03683433, NCT07304011, NCT06782542, NCT07153497). Preliminary data from a phase 1b/2 study evaluating the triple combination of VEN and ivosidenib with or without AZA in *IDH1*^mut^ ND and R/R AML have reported cCR rates of 90% in the triple combination arm versus 83% in those receiving VEN and ivosidenib, accompanied by increased MRD-negativity rates [228]. Notably, patients with signaling mutations, contributing to resistance, appeared to benefit from the triple regimen, although this result did not reach statistical significance [228]. Consequently, these combinations represent a particularly promising therapeutic approach, and forthcoming trial results will determine how to integrate them in clinical practice.

Collectively, this data suggests that combining HMAs, IDHi, and VEN, either as doublet or triplet regimens, offers a rational, biologically supported strategy to overcome IDHi resistance and improve outcomes in both ND and R/R *IDH*^mut^ AML, with ongoing trials expected to further clarify the optimal combinations and patient selection.

#### 4.2.2. Therapeutic Reinforcement: Integrating IDHi with Standard Chemotherapy

Combining IDHi with conventional chemotherapy may also help overcome resistance. It has been shown that cladribine exerts hypomethylating effects and yields superior outcomes in *IDH2*^mut^ AML patients [229]. Moreover, coadministration of daunorubicin with enasidenib was found to augment daunorubicin’s efficacy by inhibiting aldo-keto reductase 1C3 (AKR1C3), an enzyme that confers resistance to anthracyclines, while simultaneously reducing the activity of drug efflux transporters such as ATP-binding cassette subfamily B member 1 (ABCB1), ATP-binding cassette subfamily G member 2 (ABCG2), and ABCC1, which expel chemotherapeutic agents from cells [230]. Several clinical trials evaluating the combination of IDHi combined with conventional chemotherapeutic agents, both in ND and R/R *IDH*^mut^ AML, are currently ongoing (NCT02632708, NCT03825796, NCT03839771, NCT04250051, NCT04493164). Preliminary results from a study of ivosidenib with IC in ND *IDH1*^mut^ AML suggest that this combination yields durable responses, with reported CR rates of 70% and the presence of an acceptable safety profile [231].

#### 4.2.3. Blocking All Escape Routes: Pan-IDHi

A strategy to tackle isoform switching of IDH is based on the concurrent blocking of both IDH isoforms. Efficacy and safety of vorasidenib, a dual IDH1/IDH2 inhibitor, has been assessed in a phase 1 study of patients with R/R AML after IDHi failure, but the agent yielded suboptimal responses [232]. LY3410738 (crelosidenib) is another dual IDH1/IDH2 inhibitor that is currently being investigated in R/R *IDH*^mut^ AML in a phase 1 study (NCT04603001). Preliminary results suggest potent efficacy and sustained 2-HG inhibition, even in patients with prior exposure to IDHi [233]. Finally, the dual IDH1/IDH2 inhibitor HMPL-306 (ranosidenib) tested in a phase 1 study of R/R *IDH*^mut^ AML patients led to cCR rates of 34.6% and 36.4% in *IDH1*^mut^ and *IDH2*^mut^ patients, respectively [234,235]. Importantly, among those achieving CR, MRD negativity rates were 77.8% and 50%, respectively [235]. A phase 3 study of ranosidenib is currently ongoing (NCT06387069). Collectively, these data suggest that dual inhibition of IDH may offer a potential benefit in the treatment of R/R AML.

#### 4.2.4. Active-Site Targeting and PROTACs to Counteract Secondary Mutations

Overcoming resistance due to acquired mutations at the interface of the IDH dimer may be challenging. An approach to address this resistance mechanism is by employing novel inhibitors that bind to the active sites of the enzyme. HMS-101 is a unique IDH1i that binds to the active site of mutant IDH1 and has exhibited outstanding efficacy in preclinical models [236]. Another strategy with potential efficacy is the use of proteolysis targeting chimera (PROTAC) technology. PROTAC molecules can be designed to target proteins in cancer cells, such as mutant IDH, and subsequently induce their degradation [237]. Interestingly, a recent preclinical study has demonstrated degradation of mutant IDH1 in cells by employing a PROTAC-based approach that impairs STAT3 activation [238].

#### 4.2.5. Targeting Adaptive Signaling Pathways

Emerging strategies also include targeting adaptive signaling pathways that confer resistance to IDH inhibition. Considering the role of the RTK pathway in the development of resistance, administration of RTK pathway inhibitors would likely be beneficial. Inhibition of the mTOR pathway, which is highly activated in *IDH*^mut^ cells, with mTORC1 inhibitors, such as rapamycin, was effective in cells bearing *IDH1* mutations and reduced the production of 2-HG [239]. Targeting the MAPK signaling pathway may also help circumvent resistance to IDHi. Dual inhibition of IDH and MEK in mice bearing *IDH2* and *NRAS* mutations has led to a significant decrease in LSCs, providing the foundation for further evaluation of this combination in clinical trials [240]. A phase 1 study evaluating the combined use of enasidenib and cobimetinib in R/R AML patients with co-occurring *IDH2* and *RAS* mutations is underway [240]. Finally, SEL24/MEN1703 (dapolsertib), a dual PIM and FLT3 inhibitor, has demonstrated modest single-agent activity in R/R AML with *IDH1*^mut^ and *IDH2*^mut^ AML [241]. It is worth noting that responses were also observed in patients previously exposed to IDHi [241]. Altogether, this data suggests that co-targeting adaptive signaling pathways represents a rational strategy to circumvent resistance, although clinical evidence remains preliminary and further studies are needed to establish efficacy.

#### 4.2.6. Targeting DNA Repair Defects with PARP Inhibitors

IDH mutations impair cells’ ability to repair double-stranded DNA breaks during homologous recombination through 2-HG-mediated suppression of key DNA repair enzymes. These cells rely on poly(adenosine diphosphate-ribose) polymerase (PARP) enzymes to preserve genomic stability. Olaparib, a PARP inhibitor, showed efficacy in a preclinical study, even against blasts resistant to enasidenib [242]. This provided the rationale for the design of a clinical trial evaluating olaparib in patients with R/R *IDH*^mut^ AML, which is currently ongoing (NCT03953898).

#### 4.2.7. Exploiting Metabolic Vulnerabilities

Combination strategies targeting metabolic adaptations may also help circumvent IDHi inhibitor resistance. Thus, OXPHOS inhibitors or CD44 targeting could be beneficial [220,221], while targeting acetyl coenzyme A carboxylase 1 (ACC1) along with a restriction of lipid intake may offer another therapeutic option [243]. Preclinical studies have shown that *IDH1*^mut^ AML cells exhibited limited ability to grow in lipid-poor conditions and a dependency on ACC1, whose knockdown halted cell growth [243].

**Table 3:** Therapeutic strategies used to overcome resistance to IDH inhibitors in acute myeloid leukemia.

Strategy	Agent/Combination	Study Phase or Preclinical Model	Population	Clinical Trial Identifier *	Results	Ref.
**Overcoming resistance to IDH inhibitors**						
Combination regimens						
IDHi + HMAs	IVO + AZA	1/2	ND IDH1^mut^ AML	NCT06611839	Not yet recruiting	
	IVO + AZA	3b	ND IDH1^mut^ AML	NCT05907057	Recruiting	
	IVO + Gilteritinib	1	R/R AML with FLT3 + IDH mutations	NCT05756777	Recruiting	
	IVO + AZA	3	ND IDH1^mut^ AML	NCT07075016	Recruiting	
	IVO + VEN +/− AZA	1/2	ND and R/R IDH1^mut^ AML	NCT03471260	cCR: 90%	[228]
	IVO or ENA + DEC-C	1b/2	R/R IDH^mut^ AML	NCT04774393	Recruiting	
	ENA + AZA	2	R/R IDH2^mut^ AML	NCT03683433	Recruiting	
	Olutasitenib + AZA	2	R/R IDH1^mut^ AML after VEN-AZA	NCT07304011	Recruiting	
	Olutasitenib + AZA + VEN	2	ND IDH1^mut^ AML	NCT06782542	Recruiting	
	Olutasitenib + DEC-C + VEN	2	ND IDH1^mut^ AML	NCT07153497	Not yet recruiting	
IDHi + chemotherapy	IVO or ENA + IC	1	ND IDH^mut^ AML	NCT02632708	CR: 70%	[231]
	ENA + CPX-351	2	R/R IDH2^mut^ AML	NCT03825796	Active, not recruiting	
	IVO or ENA + IC	3	ND IDH^mut^ AML	NCT03839771	Active, not recruiting	
	IVO + FLAG	1	R/R IDH1^mut^ AML	NCT04250051	Active, not recruiting	
	IVO + CPX-351	2	ND IDH1^mut^ AML R/R IDH1^mut^ AML	NCT04493164	Recruiting	
Pan-IDHi						
Pan-IDHi	Vorasidenib	1	R/R IDH^mut^ AML	NCT02492737	MLS: 5.9%	
	Crelosidenib (LY3410738)	1	R/R IDH^mut^ AML	NCT04603001	Active, not recruiting	
	Ranosidenib (HMPL-306)	1	R/R IDH^mut^ AML	NCT04272957	cCR 34.6% IDH1^mut^ cCR 36.4% IDH2^mut^	[235]
	Ranosidenib	3	R/R IDH^mut^ AML	NCT06387069	Recruiting	
Active-site targeting						
Active-site targeting	HMS-101	Mouse and PDX model	IDH1^mut^ AML	NA	Induction of cellular differentiation	[236]
Targeting signaling pathways						
Targeting signaling pathways	mTORC1 inhibitors (rapamycin)	CL	IDH1^mut^ AML	NA	proliferation inhibition/ metabolic activity alteration	[239]
	ENA + cobimetinib	1	R/R IDH2^mut^ + RAS^mut^ AML	NCT05441514	Active, not recruiting	
	Dapolsertib (SEL24/MEN1703)	1/2	ND and R/R IDH^mut^ AML	NCT03008187	ORR: 9%	[241]
PARP-inhibitors	Olaparib	2	R/R IDH^mut^ AML	NCT03953898	Active, not recruiting. No results posted	
Targeting metabolic adaptations						
Targeting metabolic adaptations	OXPHOS inhibitors	PDX and PS	IDH1^mut^ AML		Improvement of IDHi efficacy	[220]
	CD44 inhibitors + IDHi	Preclinical model	IDH^mut^ AML		Combination enhances leukemia cell elimination	[221]
	ACC1 inhibitors + VEN	CL, PS	IDH^mut^ AML		Increase in sensitivity of IDH1^mut^ AML to VEN	[243]

* clinicaltrials.gov. VEN, venetoclax; AZA, azacitidine; ND, newly diagnosed; AML, acute myeloid leukemia; IC, intensive chemotherapy; R/R, relapsed/refractory; CR, complete response; cCR, composite complete response; HMA, hypomethylating agents; DEC-C, oral cedazuridine/decitabine; FLT3, FMS-like tyrosine kinase 3; MLS, morphologic leukemia-free state; NA, not applicable; CL, cell lines; PDX, patient-derived xenograft model; IDH, isocitrate dehydrogenase; IDHi, isocitrate dehydrogenase inhibitor; IVO, ivosidenib; ENA, enasidenib; FLAG, fludarabine, high dose cytarabine, and granulocyte colony-stimulating factor; mTORC1, mechanistic target of rapamycin complex 1; PARP, poly ADP ribose polymerase; OXPHOS, oxidative phosphorylation; ACC1, acetyl CoA carboxylase 1.

## 5. Menin Inhibitors

Menin inhibitors (MENINi) have emerged as one of the most promising targeted therapies for AML, particularly in genetically defined subgroups, such as patients with lysine methyltransferase 2 A (*KMT2A*, also known as *MLL*) rearrangements (*KMT2Ar*) and *NPM1* mutations. AML with *KMT2Ar* or *NPM1* mutations relies on the interaction between KMT2A and the epigenetic factor menin to maintain leukemogenic transcriptional programs [244]. By disrupting this interaction, MENINi reverse the aberrant activation of *HOX* and *MEIS1* genes, thereby inducing differentiation of leukemic cells and reducing proliferation [244].

There are currently two MENINi approved for the treatment of AML. Revumenib is approved for R/R AML with *KMT2Ar* or *NPM1* mutations, whereas ziftomenib is approved only for patients with *NPM1*-mutated R/R AML [245,246,247]. Furthermore, three other MENINi, bleximenib, enzomenib, and DS-1594, are currently being evaluated in clinical trials [245,246,247,248,249,250]. Preliminary results have shown that MENINi yield meaningful responses in R/R AML patients with KMT2Ar and *NPM1* mutations. Revumenib has demonstrated ORRs of 63.2% and 46.9% in AML patients with *KMT2Ar* and *NMP1* mutations, respectively, including heavily pretreated patients [251,252]. Similar efficacy has been demonstrated with ziftomenib, particularly in *NPM1*-mutated AML patients [253,254]. Nonetheless, a subset of patients failed to respond, while others experienced relapse after achieving an initial response, suggesting that resistance to MENINi may occur.

### 5.1. Mechanisms That Underlie Menin Inhibitor Resistance

#### 5.1.1. MEN1 Mutations

Resistance to MENINi most commonly arises through acquired mutations in the multiple endocrine neoplasia type 1 (*MEN1*) gene encoding menin that usually evolve under selective pressure from MENINi [255,256]. These mutations are somatic point mutations occurring at the menin–inhibitor interface, most frequently at residues M327, G331, S160, and T349, and disrupt MENINi binding by generating a steric clash [255,256]. However, they do not impair menin–KMT2a interaction, thereby preserving leukemogenic transcriptional programs and conferring clinical resistance [255]. These findings have also been confirmed in structural studies [256]. Importantly, while these mutations have been found in up to 39% of relapsed patients after prior revumenib treatment, they appear to occur less frequently with zifotmenib, which retains efficacy against some MEN1 mutations, such as G331R and T349, but not others, such as M327I [253,257].

#### 5.1.2. Epigenetic and Transcriptional Reprogramming

Resistance to MENINi may also arise independently of *MEN1* mutations and is potentially mediated by epigenetic mechanisms and transcriptional reprogramming. Epigenetic reactivation of noncanonical menin targets, including MYC, due to loss of polycomb repressive complex 1.1 (PRC1.1) subunits, such as polycomb group ring finger 1 (PCGF1) and BCL-6 corepressor (BCOR), represents another mechanism of MENINi resistance [258]. Of note, loss of PRC1.1 has been found to eliminate the monocytic differentiation signature in human and mouse KMT2AR-AML models, making cells sensitive to MYC and/or BCL-2 inhibition and thereby providing a rationale for combination strategies [258]. Furthermore, disrupting menin–KMT2a interaction may activate other transcriptional programs within the cell, including noncanonical lineage programs induced by the MLL3/4-UTX complex that contribute to treatment resistance [259,260].

A patient-derived xenograft model has shown that resistant cells retain suppressed expression of MESI1, and despite the successful displacement of the menin–KMT2a complex from chromatin, leukemia persists [261]. It is noteworthy that these cells lost their dependency on menin but remained dependent on the KMT2A fusion protein, thus indicating that displacing menin alone may not be sufficient to prevent the epigenetic reprogramming underlying resistance. Remarkably, inhibition of KAT6A has been shown to overcome this resistance, suggesting that KAT6A might also be implicated in driving non-genetic resistance [261].

#### 5.1.3. Co-Occurring Mutations

Finally, co-occurring mutations may contribute to the development of MENINi resistance. In particular, *FLT3*-ITD and *WT1* mutations have both been associated with resistance to MENINi in AML patients [262], with specific WT1 isoforms correlating with *HOX* overexpression [263]. However, how these mutations confer resistance remains unknown. Intriguingly, a preclinical study has demonstrated that *TP53* mutations do not mediate MENINi resistance, contrary to the contributing role of these mutations to the development of resistance to other targeted AML treatments [264].

In summary, resistance to MENINi represents a major arising challenge primarily mediated by *MEN1* mutations, as well as epigenetic and transcriptional mechanisms. However, while studies have begun to unravel potential mechanisms, further research is needed to define the exact molecular pathways driving resistance.

### 5.2. Strategies to Overcome Resistance to Menin Inhibitors

Strategies aimed at overcoming resistance to MENINi are currently under active investigation. Most approaches remain at the preclinical stage and focus on identifying resistance-associated adaptations and rational combinations to improve treatment durability. Although these findings provide important biological insights, their clinical relevance needs to be validated in prospective clinical trials. Key strategies are summarized in Table 4.

#### 5.2.1. Targeting Resistance Driven by MEN1 Mutations

In order to address resistance due to *MEN1* mutations, next-generation MENINi are under development. CHM-029 is a novel pan-MENINi designed to overcome resistant mutations with robust efficacy in preclinical AML models and retained potency against all known resistant mutations [265]. Interestingly, first-in-human clinical trials of CHM-029 are expected in the near future [265]. Moreover, switching to a different MENINi with activity against a specific *MEN1* mutation may be effective. For instance, in contrast to revumenib, ziftomenib retains activity against some *MEN1* mutations [266]. Hence, assessment of *MEN1* mutational status in patients who fail to respond or relapse is essential. Notably, switching to a different MENINi was shown to yield responses even in the absence of *MEN1* mutations in selected cases, thus highlighting that MENINi switching may be particularly promising in resistant patients [262].

#### 5.2.2. Combination Treatment with Venetoclax and Hypomethylating Agents

Combinational strategies can also be employed to circumvent resistance. The synergistic effects of MENINi and VEN have been demonstrated in AML models with *KMT2Ar* and *NPM1* mutations [266,267]. In particular, combined treatment with DS-1594b and VEN has resulted in enhanced differentiation, profoundly reduced leukemic burden, and prolonged survival compared to DS-1594b monotherapy [266]. Similar results have been observed with the combined use of another MENINi, SNDX-50469 (SNDX), and VEN [266]. Combination with HMAs may also provide benefit since MENINi disrupt leukemogenic transcriptional programs and HMAs further promote differentiation and cytotoxicity. Several ongoing clinical trials are evaluating the incorporation of MENINi into doublet and triplet regimens with VEN and HMAs in patients with both ND and R/R *KMT2Ar* or *NPM1*-mutated AML, as seen in Table 1. Preliminary results suggest outstanding efficacy of the triplet combination of ziftomenib with VEN and AZA, particularly in patients with R/R *NPM1*-mutated AML, with reported cCR rates of 80% [268]. Similar results have been observed in patients with ND *KMT2Ar* or *NPM1*-mutated AML treated with revumenib, VEN, and AZA [269]. Overall, these findings indicate that combining MENINi with VEN and AZA is a highly promising strategy. However, clinical data is still emerging and longer follow-up is needed to confirm the durability and safety of these combinations.

#### 5.2.3. Targeting MYC and Chromatin Remodeling

Preclinical data have also shown that MYC targeting can sensitize cells to MENINi [268,270]. Thus, dual targeting of the MYC/GSTP1 axis and menin with GT19715 and SNDX, respectively, has resulted in synergistic cytotoxicity in KMT2Ar AML models and has successfully overcome resistance [270]. Inhibiting chromatin remodeling complexes via inhibition of BRG1/BRM ATPases with FHD-286 has also been shown to downregulate key oncogenic transcriptional networks, including those driven by MYC, providing a rationale for combination regimens [271]. Similarly, significant MYC downregulation and enhanced apoptosis have been observed both in vivo and in vitro, with epigenetic targeting of KDM4C, a histone demethylase, using a combination of MI-503, a MENINi, and SD70, a KDM4C inhibitor [272].

#### 5.2.4. Epigenetic Co-Targeting and Emerging Agents

Several investigational agents have also shown synergy when combined with MENINi. Inhibition of LSD1 with iadademstat combined with a MENINi has exerted synergy and restored differentiation in KMT2Ar-AML preclinical models [273]. Inhibition of inosine monophosphate dehydrogenase 2 (IMPDH2), which reduces guanine nucleotides and rRNA transcription leading to reduced menin expression and subsequently impaired formation and chromatic binding of the KMT2A-fusion complex, has also displayed synergy with MENINi [274]. Other strategies with potent efficacy in preclinical models include combining MENINi with DOT1L inhibitors (pinometostat), KAT6/KAT7 acetyltransferase inhibitors, and retinoid acid receptor alpha (RARA) agonists [275,276,277]. Finally, novel treatments such as PROTACs targeting menin are currently in preclinical development [278].

**Table 4:** Therapeutic strategies used to overcome resistance to menin inhibitors in acute myeloid leukemia.

Strategy	Agent/Combination	Study Phase or Preclinical Model	Population	Clinical Trial Identifier *	Results	Ref.
**Overcoming resistance to MENINi**						
Novel MENINi	CHM-029	Preclinical model	AML		Strong efficacy	[265]
Combination regimens	Revumenib + VEN	1/2	ND NPM1^mut^ or KMT2Ar AML	NCT06284486	Recruiting	
	Revumenib + AZA + VEN	3	ND NPM1^mut^ or KMT2Ar AML	NCT06652438	Recruiting	
	Revumenib + DEC-C + VEN	1/2	ND or R/R NPM1^mut^ or KMT2Ar AML	NCT05360160	Recruiting	
	Ziftomenib + AZA + VEN	1	R/R NPM1^mut^ AML	NCT03013998	cCR: 80%	[268]
	Ziftomenib + AZA + VEN	3	ND NPM1^mut^ or KMT2Ar AML	NCT07007312	Recruiting	
MYC targeting	GT19715 + MENINi	CL	KMT2Ar AML		Highly synergistic effects	[270]
Chromatin remodeling inhibition	FHD-286 + VEN or DEC	CL and PDX	NPM1^mut^ or KMT2Ar AML		Tumor burden reduction and synergy	[271]
Histone demethylases	KDM4C + MENINi	CL	KMT2Ar AML		Synergistic effects	[272]
Inhibition of LSD1	LSD1 inhibitor + MENINi	PDX	KMT2Ar AML		Marked reduction in leukemic burden	[273]
	IMPDH2i + MENINi	CL and PDX	KMT2Ar AML		Synergistic effects	[274]
DOT1L	Pinometostat + revumenib	CL	KMT2Ar AML		Modest responses	[275]
Other agents	KAT6/KAT7 acetyltransferase inhibitors + MENINi	CL	KMT2Ar AML		Represses oncogenic transcription and overcomes resistance	[276]
	RARA agonists + revumenib	CL	KMT2Ar and NPM1^mut^ AML		Synergistic induction of differentiation or apoptosis	[277]

* clinicaltrials.gov. VEN, venetoclax; AZA, azacitidine; ND, newly diagnosed; AML, acute myeloid leukemia; R/R, relapsed/refractory; cCR, composite complete response; DEC, decitabine; DEC-C, oral cedazuridine/decitabine; LSD1; lysine-specific histone demethylase 1A; CL, cell lines; PDX, patient-derived xenograft model; MENINi, menin inhibitor; NPM1^mut^, nucleophosmin-mutated; KMT2Ar, histone–lysine N-methyltransferase 2A rearranged; KDM4C, lysine demethylase 4C; IMPDH2i, inosine-5′-monophosphate dehydrogenase 2 inhibitor; DOT1L, disruptor of telomeric silencing 1-Like; RARA, retinoid acid receptor alpha.

## 6. Conclusions

The development of novel treatments targeting BCL-2, FLT3, IDH1/2, and menin has reshaped the treatment landscape of AML, demonstrating that certain types of leukemia can be durably controlled through pathway-directed inhibition. However, the emergence of both intrinsic and acquired resistance remains a major limitation concerning the long-term efficacy of these agents. Several mechanisms, such as lineage plasticity, acquired mutations, activation of bypass signaling pathways, epigenetic alterations, and metabolic rewiring, are implicated in the development of resistance, underscoring the biological heterogeneity and dynamic adaptability of AML and highlighting that inhibition with single agents rarely leads to disease cure.

Despite these challenges, employment of targeted agents in AML treatment has been met with clinical success. The utilization of combination regimens and earlier deployment of these agents in treatment-naïve settings may improve outcomes. As the mechanisms underlying resistance become better defined, precision-guided and adaptive therapeutic strategies may improve clinical outcomes.

## 7. Future Directions

Overcoming treatment resistance in AML will require integration of mechanistic biology with innovative treatment approaches. Combination regimens targeting cooperating vulnerabilities, such as the concurrent use of VEN with IDHi or MENINi, have already shown encouraging results and should be investigated rigorously across molecular subtypes. Targeting the BM microenvironment, chromatin remodeling, and epigenetic targeting emerge as promising approaches. The integration of targeted agents with immune-based approaches may also offer opportunities to overcome resistance via the multi-axis blockade of leukemic survival. MRD-driven adaptive therapy switching combined with single-cell monitoring and other emerging disease surveillance technologies are near-term testable priorities that will enable the early detection of resistant subpopulations at subclinical levels, thereby allowing timely, pre-emptive treatment modification and supporting clinical decision making. Leveraging these approaches will probably help to produce durable remissions in AML and ultimately eliminate resistant disease.

## Data Availability

The data that support the findings of this study are available from the corresponding author, upon reasonable request.

## References

[1] Döhner H., DiNardo C.D., Appelbaum F.R., Craddock C., Dombret H., Ebert B.L., Fenaux P., Godley L.A., Hasserjian R.P., Larson R.A. (2024). Genetic risk classification for adults with AML receiving less-intensive therapies: The 2024 ELN recommendations. Blood.

[2] Shimony S., Stahl M., Stone R.M. (2025). Acute Myeloid Leukemia: 2025 Update on Diagnosis, Risk-Stratification, and Management. Am. J. Hematol..

[3] Stafylidis C., Vlachopoulou D., Kontandreopoulou C.N., Diamantopoulos P.T. (2024). Unmet Horizons: Assessing the Challenges in the Treatment of TP53-Mutated Acute Myeloid Leukemia. J. Clin. Med..

[4] Schmalbrock L.K., Dolnik A., Cocciardi S., Sträng E., Theis F., Jahn N., Panina E., Blätte T.J., Herzig J., Skambraks S. (2021). Clonal Evolution of Acute Myeloid Leukemia with FLT3-ITD Mutation Under Treatment With Midostaurin. Blood.

[5] Markouli M., Pagoni M.N., Diamantopoulos P. (2025). BCL-2 inhibitors in hematological malignancies: Biomarkers that predict response and management strategies. Front. Oncol..

[6] Qureshi Z., Jamil A., Altaf F., Siddique R.M. (2025). Meta-analysis of Therapeutic Approaches in Acute Myeloid Leukemia: Unveiling Trends and Predictors of Treatment Response. Am. J. Clin. Oncol..

[7] Thol F.R., Döhner H., Ganser A. (2024). How I treat refractory and relapsed acute myeloid leukemia. Blood.

[8] Delbridge A.R., Strasser A. (2015). The BCL-2 protein family, BH3-mimetics and cancer therapy. Cell Death Differ..

[9] Singh R., Letai A., Sarosiek K. (2019). Regulation of apoptosis in health and disease: The balancing act of BCL-2 family proteins. Nat. Rev. Mol. Cell Biol..

[10] Tsujimoto Y., Finger L.R., Yunis J., Nowell P.C., Croce C.M. (1984). Cloning of the chromosome breakpoint of neoplastic B cells with the t(14;18) chromosome translocation. Science.

[11] Pepper C., Hoy T., Bentley P. (1998). Elevated Bcl-2/Bax are a consistent feature of apoptosis resistance in B-cell chronic lymphocytic leukaemia and are correlated with in vivo chemoresistance. Leuk. Lymphoma.

[12] Yunis J.J., Mayer M.G., Arnesen M.A., Aeppli D.P., Oken M.M., Frizzera G. (1989). *bcl-2* and other genomic alterations in the prognosis of large-cell lymphoma. N. Engl. J. Med..

[13] Campos L., Rouault J.P., Sabido O., Oriol P., Roubi N., Vasselon C., Archimbaud E., Magaud J.P., Guyotat D. (1993). High expression of bcl-2 protein in acute myeloid leukemia cells is associated with poor response to chemotherapy. Blood.

[14] https://www.fda.gov/drugs/resources-information-approved-drugs/fda-approves-venetoclax-cll-and-sll.

[15] https://www.fda.gov/drugs/resources-information-approved-drugs/fda-grants-regular-approval-venetoclax-combination-untreated-acute-myeloid-leukemia.

[16] DiNardo C.D., Jonas B.A., Pullarkat V., Thirman M.J., Garcia J.S., Wei A.H., Konopleva M., Döhner H., Letai A., Fenaux P. (2020). Azacitidine and Venetoclax in Previously Untreated Acute Myeloid Leukemia. N. Engl. J. Med..

[17] Pratz K.W., Jonas B.A., Pullarkat V., Thirman M.J., Garcia J.S., Döhner H., Récher C., Fiedler W., Yamamoto K., Wang J. (2024). Long-term follow-up of VIALE-A: Venetoclax and azacitidine in chemotherapy-ineligible untreated acute myeloid leukemia. Am. J. Hematol..

[18] Konopleva M., Pollyea D.A., Potluri J., Chyla B., Hogdal L., Busman T., McKeegan E., Salem A.H., Zhu M., Ricker J.L. (2016). Efficacy and Biological Correlates of Response in a Phase II Study of Venetoclax Monotherapy in Patients with Acute Myelogenous Leukemia. Cancer Discov..

[19] Lin K.H., Winter P.S., Xie A., Roth C., Martz C.A., Stein E.M., Anderson G.R., Tingley J.P., Wood K.C. (2016). Targeting MCL-1/BCL-XL Forestalls the Acquisition of Resistance to ABT-199 in Acute Myeloid Leukemia. Sci. Rep..

[20] Pan R., Hogdal L.J., Benito J.M., Bucci D., Han L., Borthakur G., Cortes J., DeAngelo D.J., Debose L., Mu H. (2014). Selective BCL-2 inhibition by ABT-199 causes on-target cell death in acute myeloid leukemia. Cancer Discov..

[21] Xiang Z., Luo H., Payton J.E., Cain J., Ley T.J., Opferman J.T., Tomasson M.H. (2010). Mcl1 haploinsufficiency protects mice from Myc-induced acute myeloid leukemia. J. Clin. Investig..

[22] Glaser S.P., Lee E.F., Trounson E., Bouillet P., Wei A., Fairlie W.D., Izon D.J., Zuber J., Rappaport A.R., Herold M.J. (2012). Anti-apoptotic Mcl-1 is essential for the development and sustained growth of acute myeloid leukemia. Genes. Dev..

[23] Kuusanmäki H., Leppä A.M., Pölönen P., Kontro M., Dufva O., Deb D., Yadav B., Brück O., Kumar A., Everaus H. (2020). Phenotype-based drug screening reveals association between venetoclax response and differentiation stage in acute myeloid leukemia. Haematologica.

[24] Zhang H., Nakauchi Y., Köhnke T., Stafford M., Bottomly D., Thomas R., Wilmot B., McWeeney S.K., Majeti R., Tyner J.W. (2020). Integrated analysis of patient samples identifies biomarkers for venetoclax efficacy and combination strategies in acute myeloid leukemia. Nat. Cancer.

[25] Pei S., Pollyea D.A., Gustafson A., Stevens B.M., Minhajuddin M., Fu R., Riemondy K.A., Gillen A.E., Sheridan R.M., Kim J. (2020). Monocytic Subclones Confer Resistance to Venetoclax-Based Therapy in Patients with Acute Myeloid Leukemia. Cancer Discov..

[26] Allen B., Bottomly D., Köhnke T., Wang A., Lin H.Y., Johnson K., Kenna I., Streltsova A., Martin E., Chen R. (2025). A CEBPB/IL-1β/TNF-α feedback loop drives drug resistance to venetoclax and MDM2 inhibitors in monocytic leukemia. Blood.

[27] Kuusanmäki H., Dufva O., Vähä-Koskela M., Leppä A.M., Huuhtanen J., Vänttinen I., Nygren P., Klievink J., Bouhlal J., Pölönen P. (2023). Erythroid/megakaryocytic differentiation confers BCL-XL dependency and venetoclax resistance in acute myeloid leukemia. Blood.

[28] Lagadinou E.D., Sach A., Callahan K., Rossi R.M., Neering S.J., Minhajuddin M., Ashton J.M., Pei S., Grose V., O’Dwyer K.M. (2013). BCL-2 inhibition targets oxidative phosphorylation and selectively eradicates quiescent human leukemia stem cells. Cell Stem Cell.

[29] Pollyea D.A., Stevens B.M., Jones C.L., Winters A., Pei S., Minhajuddin M., D’Alessandro A., Culp-Hill R., Riemondy K.A., Gillen A.E. (2018). Venetoclax with azacitidine disrupts energy metabolism and targets leukemia stem cells in patients with acute myeloid leukemia. Nat. Med..

[30] Roca-Portoles A., Rodriguez-Blanco G., Sumpton D., Cloix C., Mullin M., Mackay G.M., O’Neill K., Lemgruber L., Luo X., Tait S.W.G. (2020). Venetoclax causes metabolic reprogramming independent of BCL-2 inhibition. Cell Death Dis..

[31] Stevens B.M., Jones C.L., Pollyea D.A., Culp-Hill R., D’Alessandro A., Winters A., Krug A., Abbott D., Goosman M., Pei S. (2020). Fatty acid metabolism underlies venetoclax resistance in acute myeloid leukemia stem cells. Nat. Cancer.

[32] Escudero S., Zaganjor E., Lee S., Mill C.P., Morgan A.M., Crawford E.B., Chen J., Wales T.E., Mourtada R., Luccarelli J. (2018). Dynamic Regulation of Long-Chain Fatty Acid Oxidation by a Noncanonical Interaction between the MCL-1 BH3 Helix and VLCAD. Mol. Cell.

[33] Jones C.L., Stevens B.M., Pollyea D.A., Culp-Hill R., Reisz J.A., Nemkov T., Gehrke S., Gamboni F., Krug A., Winters A. (2020). Nicotinamide Metabolism Mediates Resistance to Venetoclax in Relapsed Acute Myeloid Leukemia Stem Cells. Cell Stem Cell.

[34] Ebner J., Schmoellerl J., Piontek M., Manhart G., Troester S., Carter B.Z., Neubauer H., Moriggl R., Szakács G., Zuber J. (2023). ABCC1 and glutathione metabolism limit the efficacy of BCL-2 inhibitors in acute myeloid leukemia. Nat. Commun..

[35] Zhang Q., Riley-Gillis B., Han L., Jia Y., Lodi A., Zhang H., Ganesan S., Pan R., Konoplev S.N., Sweeney S.R. (2022). Activation of RAS/MAPK pathway confers MCL-1 mediated acquired resistance to BCL-2 inhibitor venetoclax in acute myeloid leukemia. Signal Transduct. Target. Ther..

[36] Alkhatabi H.A., Zohny S.F., Mohammed M.R.S., Choudhry H., Rehan M., Ahmad A., Ahmed F., Khan M.I. (2022). Venetoclax-Resistant MV4-11 Leukemic Cells Activate PI3K/AKT Pathway for Metabolic Reprogramming and Redox Adaptation for Survival. Antioxidants.

[37] Chong S.J.F., Lai J.X.H., Iskandar K., Leong B.J., Wang C., Wang Y., Guièze R., Raman D., Lim R.H.F., Wu C.J. (2025). Superoxide-mediated phosphorylation and stabilization of Mcl-1 by AKT underlie venetoclax resistance in hematologic malignancies. Leukemia.

[38] Hyun S.Y., Na E.J., Kim Y.R., Min Y.H., Cheong J.-W. (2025). Na^+^/H^+^ Exchanger 1 Inhibition Overcomes Venetoclax Resistance in Acute Myeloid Leukemia. Cells.

[39] Guièze R., Liu V.M., Rosebrock D., Hernández-Sánchez M., Martinez Zurita A., Sun J., Ten Hacken E., Baranowski K., Thompson P.A., Heo J.M. (2019). Mitochondrial Reprogramming Underlies Resistance to BCL-2 Inhibition in Lymphoid Malignancies. Cancer Cell.

[40] Chen X., Glytsou C., Zhou H., Narang S., Reyna D.E., Lopez A., Sakellaropoulos T., Gong Y., Kloetgen A., Yap Y.S. (2019). Targeting Mitochondrial Structure Sensitizes Acute Myeloid Leukemia to Venetoclax Treatment. Cancer Discov..

[41] Glytsou C., Chen X., Zacharioudakis E., Al-Santli W., Zhou H., Nadorp B., Lee S., Lasry A., Sun Z., Papaioannou D. (2023). Mitophagy Promotes Resistance to BH3 Mimetics in Acute Myeloid Leukemia. Cancer Discov..

[42] Blombery P., Anderson M.A., Gong J.N., Thijssen R., Birkinshaw R.W., Thompson E.R., Teh C.E., Nguyen T., Xu Z., Flensburg C. (2019). Acquisition of the Recurrent Gly101Val Mutation in BCL2 Confers Resistance to Venetoclax in Patients with Progressive Chronic Lymphocytic Leukemia. Cancer Discov..

[43] Moujalled D.M., Brown F.C., Chua C.C., Dengler M.A., Pomilio G., Anstee N.S., Litalien V., Thompson E., Morley T., MacRaild S. (2023). Acquired mutations in BAX confer resistance to BH3-mimetic therapy in acute myeloid leukemia. Blood.

[44] Kim K., Maiti A., Loghavi S., Pourebrahim R., Kadia T.M., Rausch C.R., Furudate K., Daver N.G., Alvarado Y., Ohanian M. (2021). Outcomes of TP53-mutant acute myeloid leukemia with decitabine and venetoclax. Cancer.

[45] Aldoss I., Zhang J., Pillai R., Shouse G., Sanchez J.F., Mei M., Nakamura R., Stein A.S., Forman S.J., Marcucci G. (2019). Venetoclax and hypomethylating agents in TP53-mutated acute myeloid leukaemia. Br. J. Haematol..

[46] DiNardo C.D., Tiong I.S., Quaglieri A., MacRaild S., Loghavi S., Brown F.C., Thijssen R., Pomilio G., Ivey A., Salmon J.M. (2020). Molecular patterns of response and treatment failure after frontline venetoclax combinations in older patients with AML. Blood.

[47] Thijssen R., Diepstraten S.T., Moujalled D., Chew E., Flensburg C., Shi M.X., Dengler M.A., Litalien V., MacRaild S., Chen M. (2021). Intact TP-53 function is essential for sustaining durable responses to BH3-mimetic drugs in leukemias. Blood.

[48] Nechiporuk T., Kurtz S.E., Nikolova O., Liu T., Jones C.L., D’Alessandro A., Culp-Hill R., d’Almeida A., Joshi S.K., Rosenberg M. (2019). The TP53 Apoptotic Network Is a Primary Mediator of Resistance to BCL2 Inhibition in AML Cells. Cancer Discov..

[49] Fischer M. (2017). Census and evaluation of p53 target genes. Oncogene.

[50] Chyla B., Daver N., Doyle K., McKeegan E., Huang X., Ruvolo V., Wang Z., Chen K., Souers A., Leverson J. (2018). Genetic Biomarkers Of Sensitivity and Resistance to Venetoclax Monotherapy in Patients With Relapsed Acute Myeloid Leukemia. Am. J. Hematol..

[51] Mali R.S., Zhang Q., DeFilippis R., Cavazos A., Kuruvilla V.M., Raman J., Mody V., Choo E.F., Dail M., Shah N.P. (2021). Venetoclax combines synergistically with FLT3 inhibition to effectively target leukemic cells in FLT3-ITD+ acute myeloid leukemia models. Haematologica.

[52] Yoshimoto G., Miyamoto T., Jabbarzadeh-Tabrizi S., Iino T., Rocnik J.L., Kikushige Y., Mori Y., Shima T., Iwasaki H., Takenaka K. (2009). FLT3-ITD up-regulates MCL-1 to promote survival of stem cells in acute myeloid leukemia via FLT3-ITD-specific STAT5 activation. Blood.

[53] Shu W., Yang Q., He D., Li Y., Le J., Cai Q., Dai H., Luo L., Chen B., Gong Y. (2025). Impact of KIT mutation on efficacy of venetoclax and hypomethylating agents in newly diagnosed acute myeloid leukemia. Eur. J. Med. Res..

[54] Wang L., Gao H., Fu Q., Jiang Q., Jiang H., Wang Y., Xu L., Zhang X., Huang X., Tang F. (2025). Clinical and Molecular Predictors of Response and Survival Following Venetoclax Plus Hypomethylating Agents in Relapsed/Refractory Acute Myeloid Leukemia: A Single-Center Study in Chinese Patients. Cancers.

[55] Bewersdorf J.P., Shimony S., Shallis R.M., Liu Y., Berton G., Schaefer E.J., Zeidan A.M., Goldberg A., Stein E., Marcucci G. (2024). Combination therapy with hypomethylating agents and venetoclax versus intensive induction chemotherapy in IDH1- or IDH2-mutant newly diagnosed acute myeloid leukemia-A multicenter cohort study. Am. J. Hematol..

[56] Berton G., Sedaki B., Collomb E., Benachour S., Loschi M., Mohty B., Saillard C., Hicheri Y., Rouzaud C., Maisano V. (2024). Poor prognosis of SRSF2 gene mutations in patients treated with VEN-AZA for newly diagnosed acute myeloid leukemia. Leuk. Res..

[57] Rahmani N.E., Ramachandra N., Sahu S., Gitego N., Lopez A., Pradhan K., Bhagat T.D., Gordon-Mitchell S., Pena B.R., Kazemi M. (2021). ASXL1 mutations are associated with distinct epigenomic alterations that lead to sensitivity to venetoclax and azacytidine. Blood Cancer J..

[58] Brunetti L., Gundry M.C., Sorcini D., Guzman A.G., Huang Y.H., Ramabadran R., Gionfriddo I., Mezzasoma F., Milano F., Nabet B. (2018). Mutant NPM1 Maintains the Leukemic State through HOX Expression. Cancer Cell.

[59] Kontro M., Kumar A., Majumder M.M., Eldfors S., Parsons A., Pemovska T., Saarela J., Yadav B., Malani D., Fløisand Y. (2017). HOX gene expression predicts response to BCL-2 inhibition in acute myeloid leukemia. Leukemia.

[60] Schoenwaelder S.M., Jarman K.E., Gardiner E.E., Hua M., Qiao J., White M.J., Josefsson E.C., Alwis I., Ono A., Willcox A. (2011). Bcl-xL-inhibitory BH3 mimetics can induce a transient thrombocytopathy that undermines the hemostatic function of platelets. Blood.

[61] Stein A.S., Zhang J., Ghoda L.Y., Robbins M., Croslin C., Sinaki B., Aribi A., Blackmon D.O.A., Pourhassan H., Ball B.J. (2024). Phase 1 Trial of Navitoclax/Venetoclax/Decitabine Combination in Relapsed/Refractory (R/R) Acute Myeloid Leukemia (AML). Blood.

[62] Balachander S.B., Criscione S.W., Byth K.F., Cidado J., Adam A., Lewis P., Macintyre T., Wen S., Lawson D., Burke K. (2020). AZD4320, A Dual Inhibitor of Bcl-2 and Bcl-xL, Induces Tumor Regression in Hematologic Cancer Models without Dose-limiting Thrombocytopenia. Clin. Cancer Res..

[63] Marconi G., Arslan S., Fleming S., Curti A., SBlachly J., Martinelli G., Giovanni Della Porta M., Bajel A., Sauer T., Crysandt M. (2023). Safety and Tolerability of AZD0466 As Monotherapy for Patients with Advanced Hematologic Malignancies. Results from a Phase I/II Trial. Blood.

[64] Kadia T., Kropf P., Leahy M., Chua C.C., Fleming S., Oliai C., Kwan J., Fong C.Y., Richmond J., Canell P. (2025). Results of the APG2575AU101 Study of lisaftoclax (APG-2575) combined with azacitidine (AZA) in patients with newly diagnosed (ND) or prior venetoclax–exposed myeloid malignancies. Blood.

[65] Kotschy A., Szlavik Z., Murray J., Davidson J., Maragno A.L., Le Toumelin-Braizat G., Chanrion M., Kelly G.L., Gong J.N., Moujalled D.M. (2016). The MCL1 inhibitor S63845 is tolerable and effective in diverse cancer models. Nature.

[66] Brennan M.S., Chang C., Tai L., Lessene G., Strasser A., Dewson G., Kelly G.L., Herold M.J. (2018). Humanized Mcl-1 mice enable accurate preclinical evaluation of MCL-1 inhibitors destined for clinical use. Blood.

[67] Szlavik Z., Csekei M., Paczal A., Szabo Z.B., Sipos S., Radics G., Proszenyak A., Balint B., Murray J., Davidson J. (2020). Discovery of S64315, a potent and selective Mcl-1 inhibitor. J. Med. Chem..

[68] Gill H., Moors I., Porkka K., Moshe Y., Yoon S.S., Daver N., Ocio E.M., Fukaya M., Sauer T., Mateos M.V. (2024). A First in Human Study of VOB560 in Combination with MIK665 in Patients with Relapsed/Refractory Non-Hodgkin Lymphoma, Acute Myeloid Leukemia, or Multiple Myeloma. Blood.

[69] Wang X., Bathina M., Lynch J., Koss B., Calabrese C., Frase S., Schuetz J.D., Rehg J.E., Opferman J.T. (2013). Deletion of MCL-1 causes lethal cardiac failure and mitochondrial dysfunction. Genes Dev..

[70] Tron A.E., Belmonte M.A., Adam A., Aquila B.M., Boise L.H., Chiarparin E., Cidado J., Embrey K.J., Gangl E., Gibbons F.D. (2018). Discovery of Mcl-1-specific inhibitor AZD5991 and preclinical activity in multiple myeloma and acute myeloid leukemia. Nat. Commun..

[71] Desai P., Lonial S., Cashen A., Kamdar M., Flinn I., O’Brien S., Garcia J.S., Korde N., Moslehi J., Wey M. (2024). A Phase 1 First-in-Human Study of the MCL-1 Inhibitor AZD5991 in Patients with Relapsed/Refractory Hematologic Malignancies. Clin. Cancer Res..

[72] Jin S., Cojocari D., Purkal J.J., Popovic R., Talaty N.N., Xiao Y., Solomon L.R., Boghaert E.R., Leverson J.D., Phillips D.C. (2020). 5-Azacitidine Induces NOXA to Prime AML Cells for Venetoclax-Mediated Apoptosis. Clin. Cancer Res..

[73] Tibes R., Bogenberger J.M. (2019). Transcriptional Silencing of MCL-1 Through Cyclin-Dependent Kinase Inhibition in Acute Myeloid Leukemia. Front. Oncol..

[74] Bogenberger J., Whatcott C., Hansen N., Delman D., Shi C.X., Kim W., Haws H., Soh K., Lee Y.S., Peterson P. (2017). Combined venetoclax and alvocidib in acute myeloid leukemia. Oncotarget.

[75] Jonas B.A., Hou J., Roboz G.J., Alvares C.L., Jeyakumar D., Edwards J.R., Erba H.P., Kelly R.J., Röllig C., Fiedler W. (2023). A phase 1b study of venetoclax and alvocidib in patients with relapsed/refractory acute myeloid leukemia. Hematol. Oncol..

[76] Knorr K.L.B., Schneider P.A., Meng X.W., Dai H., Smith B.D., Hess A.D., Karp J.E., Kaufmann S.H. (2015). MLN4924 induces Noxa upregulation in acute myelogenous leukemia and synergizes with Bcl-2 inhibitors. Cell Death Differ..

[77] Murthy G.S.G., Saliba A.N., Szabo A., Harrington A., Abedin S., Carlson K., Michaelis L., Runaas L., Baim A., Hinman A. (2024). A phase I study of pevonedistat, azacitidine, and venetoclax in patients with relapsed/refractory acute myeloid leukemia. Haematologica.

[78] Short N.J., Wierzbowska A., Cluzeau T., Laribi K., Recher C., Czyz J., Ochrem B., Ades L., Gallego-Hernanz M.P., Heiblig M. (2025). Azacitidine and venetoclax with or without pevonedistat in patients with newly diagnosed acute myeloid leukemia. Leuk. Lymphoma.

[79] Luedtke D.A., Su Y., Liu S., Edwards H., Wang Y., Lin H., Taub J.W., Ge Y. (2018). Inhibition of XPO1 enhances cell death induced by ABT-199 in acute myeloid leukaemia via mcl-1. J. Cell Mol. Med..

[80] Xing L., Guo Z., Huang C., Jing H., Qiao S. (2024). The Efficacy and Safety of the Combination Therapy of Decitabine, Venetoclax, and Selinexor (DVS) for Newly Diagnosed Unfit AML and MDS, and Relapsed/ Refractory AML and High-Risk MDS—A Multicenter, Single Arm, Prospective Clinical Study. Blood.

[81] Ball S., Byrne M.T., Fischer M.A., Awan F.T., Tomlinson B.K., Stopczynski T., Patil L., Gu C.J., Leonardi C., Walsh K. (2025). AML-117: A Phase 1b Study of Selinexor and Venetoclax in Patients With Relapsed or Refractory Acute Myeloid Leukemia. Clin. Lymphoma Myeloma Leuk..

[82] Han L., Zhang Q., Dail M., Shi C., Cavazos A., Ruvolo V.R., Zhao Y., Kim E., Rahmani M., Mak D. (2020). Concomitant targeting of BCL2 with venetoclax and MAPK signaling with cobimetinib in acute myeloid leukemia models. Haematologica.

[83] Khurana A., Shafer D.A. (2019). MDM2 antagonists as a novel treatment option for acute myeloid leukemia: Perspectives on the therapeutic potential of idasanutlin (RG7388). OncoTargets Ther..

[84] Konopleva M.Y., Dail M., Daver N.G., Garcia J.S., Jonas B.A., Yee K.W.L., Kelly K.R., Vey N., Assouline S., Roboz G.J. (2024). Venetoclax and Cobimetinib in Relapsed/Refractory AML: A Phase 1b Trial. Clin. Lymphoma Myeloma Leuk..

[85] Daver N.G., Dail M., Garcia J.S., Jonas B.A., Yee K.W.L., Kelly K.R., Vey N., Assouline S., Roboz G.J., Paolini S. (2023). Venetoclax and idasanutlin in relapsed/refractory AML: A nonrandomized, open-label phase 1b trial. Blood.

[86] Garg R., Allen K.J.H., Dawicki W., Geoghegan E.M., Ludwig D.L., Dadachova E. (2021). 225Ac-labeled CD33-targeting antibody reverses resistance to Bcl-2 inhibitor venetoclax in acute myeloid leukemia models. Cancer Med..

[87] Ureshino H., Ueshima T., Yamaguchi T., Takashima M., Sanuki Y., Ichinohe T. (2025). (R)-WAC-224, a new anticancer quinolone, combined with venetoclax and azacitidine overcomes venetoclax-resistant AML through MCL-1 downregulation. Sci. Rep..

[88] Mino T., Ureshino H., Ueshima T., Kashimoto N., Yamaguchi T., Naka K., Inaba T., Ichinohe T. (2023). (R)-Wac-224 Monotherapy or Combination with Cytarabine/Venetoclax Confers Promising Activities Against Acute Myeloid Leukemia. Blood.

[89] Lewis A.C., Pope V.S., Tea M.N., Li M., Nwosu G.O., Nguyen T.M., Wallington-Beddoe C.T., Moretti P.A.B., Anderson D., Creek D.J. (2022). Ceramide-induced integrated stress response overcomes Bcl-2 inhibitor resistance in acute myeloid leukemia. Blood.

[90] Sharon D., Cathelin S., Mirali S., Di Trani J.M., Yanofsky D.J., Keon K.A., Rubinstein J.L., Schimmer A.D., Ketela T., Chan S.M. (2019). Inhibition of mitochondrial translation overcomes venetoclax resistance in AML through activation of the integrated stress response. Sci. Transl. Med..

[91] Singh S., Gomez H.J., Thakkar S., Singh S.P., Parihar A.S. (2023). Overcoming Acquired Drug Resistance to Cancer Therapies through Targeted STAT3 Inhibition. Int. J. Mol. Sci..

[92] Martínez-López J., Montesinos P., López-Muñoz N., Ayala R., Martínez-Sánchez P., Gorrochategui J., Rojas-Rudilla J.L., Primo D., Bergua-Burgues J.M., Calbacho M. (2022). Biomarker-driven phase Ib clinical trial of OPB-111077 in acute myeloid leukemia. Med. Int..

[93] Shastri A., Goldfinger M., Mantzaris I., Garcia-Moreno G., Kadia T.M., Ning J., Tirone B., Gandhi R., Ravandi F., Pemmaraju N. (2024). A Phase 1 Study Investigating the Safety and Efficacy of Danvatirsen As Monotherapy Followed By Combination with Venetoclax in Patients with Relapsed/Refractory MDS and AML. Blood.

[94] Borate U.M., Madanat Y.F., Tognon C., Mishra S., Kaempf A., Patel P.A., Kurtz S.E., Johnson K., Vasu S., Baker S.D. (2023). Results of a Phase 1 Trial Testing the Novel Combination Therapy of Venetoclax and Ruxolitinib in Relapsed/Refractory Acute Myeloid Leukemia Patients. Blood.

[95] Zhang L., Kang H., Valerio M., Hoang D.H., Pathak K., Garcia-Mansfield K., Lu X., Guo W., Fu Y.H., He X. (2025). Pharmacological Inhibition of miR-126 Enhances Venetoclax Activity in Acute Myeloid Leukemia. Blood.

[96] Buono R., Juarez D., Paul M., Skuli S.J., Wong I.B., Tarnekar I., Ying Z., Le I., Wertheim G., Bakayoko A. (2025). Pitavastatin counteracts venetoclax resistance mechanisms in acute myeloid leukemia by depleting geranylgeranyl pyrophosphate. bioRxiv.

[97] Sanchez J.R., Liu C., Pawar V., Huang Y., Diaz-Rohena D., Singh J., Koppikar P., Lai T.H., St John L., Puduvalli V. (2026). NAMPT inhibition uncovers therapeutic vulnerabilities to venetoclax and chemotherapy in acute myelogenous leukemia. Leuk. Lymphoma.

[98] de Lacerda M.P., Nunes E., Steffenello G., Carneiro T.X., Boettcher I.S., Dall’Oglio A.C., Martini F., Esmeraldino L., Molossi M., Wagner A.O.M. (2024). Safety and Efficacy of Venetoclax, Cytarabine and Metformin for Relapsed-Refractory and Induction-Ineligible Acute Myeloid Leukemia. Blood.

[99] Garcia-Manero G., McCloskey J., Griffiths E.A., Yee K.W.L., Zeidan A.M., Al-Kali A., Deeg H.J., Patel P.A., Sabloff M., Keating M.M. (2024). Oral decitabine-cedazuridine versus intravenous decitabine for myelodysplastic syndromes and chronic myelomonocytic leukaemia (ASCERTAIN): A registrational, randomised, crossover, pharmacokinetics, phase 3 study. Lancet Haematol..

[100] Levitz D., Saunthararajah Y., Fedorov K., Shapiro L.C., Mantzaris I., Shastri A., Kornblum N., Sica R.A., Shah N., Konopleva M. (2023). A Metabolically Optimized, Noncytotoxic Low-Dose Weekly Decitabine/Venetoclax in MDS and AML. Clin. Cancer Res..

[101] Salamero O., Montesinos P., Willekens C., Pérez-Simón J.A., Pigneux A., Récher C., Popat R., Carpio C., Molinero C., Mascaró C. (2020). First-in-Human Phase I Study of Iadademstat (ORY-1001): A First-in-Class Lysine-Specific Histone Demethylase 1A Inhibitor, in Relapsed or Refractory Acute Myeloid Leukemia. J. Clin. Oncol..

[102] Maes T., Mascaró C., Tirapu I., Estiarte A., Ciceri F., Lunardi S., Guibourt N., Perdones A., Lufino M.M.P., Somervaille T.C.P. (2018). ORY-1001, a Potent and Selective Covalent KDM1A Inhibitor, for the Treatment of Acute Leukemia. Cancer Cell.

[103] Wang B.R., Wan C.L., Liu S.B., Qiu Q.C., Wu T.M., Wang J., Li Y.Y., Ge S.S., Qiu Y., Shen X.D. (2021). A Combined Histone Deacetylases Targeting Strategy to Overcome Venetoclax Plus Azacitidine Regimen Resistance in Acute Myeloid Leukaemia: Three Case Reports. Front. Oncol..

[104] Desikan S.P., Ravandi F., Pemmaraju N., Konopleva M., Loghavi S., Jabbour E.J., Daver N., Jain N., Chien K.S., Maiti A. (2022). A Phase II Study of Azacitidine, Venetoclax, and Trametinib in Relapsed or Refractory Acute Myeloid Leukemia Harboring RAS Pathway-Activating Mutations. Acta Haematol..

[105] Lavagna-Sévenier C., Marchetto S., Birnbaum D., Rosnet O. (1998). FLT3 signaling in hematopoietic cells involves CBL, SHC and an unknown P115 as prominent tyrosine-phosphorylated substrates. Leukemia.

[106] Gilliland D.G., Griffin J.D. (2002). The roles of FLT3 in hematopoiesis and leukemia. Blood.

[107] Takahashi S. (2011). Downstream molecular pathways of FLT3 in the pathogenesis of acute myeloid leukemia: Biology and therapeutic implications. J. Hematol. Oncol..

[108] Yokota S., Kiyoi H., Nakao M., Iwai T., Misawa S., Okuda T., Sonoda Y., Abe T., Kahsima K., Matsuo Y. (1997). Internal tandem duplication of the FLT3 gene is preferentially seen in acute myeloid leukemia and myelodysplastic syndrome among various hematological malignancies. A study on a large series of patients and cell lines. Leukemia.

[109] Yanada M., Matsuo K., Suzuki T., Kiyoi H., Naoe T. (2005). Prognostic significance of FLT3 internal tandem duplication and tyrosine kinase domain mutations for acute myeloid leukemia: A meta-analysis. Leukemia.

[110] Song M.K., Park B.B., Uhm J.E. (2022). Clinical Efficacies of FLT3 Inhibitors in Patients with Acute Myeloid Leukemia. Int. J. Mol. Sci..

[111] https://aml-hub.com/medical-information/quizartinib-granted-fda-approval-for-patients-with-newly-diagnosed-flt3-itd-mutated-aml.

[112] Stone R.M., Mandrekar S.J., Sanford B.L., Laumann K., Geyer S., Bloomfield C.D., Thiede C., Prior T.W., Döhner K., Marcucci G. (2017). Midostaurin plus Chemotherapy for Acute Myeloid Leukemia with a FLT3 Mutation. N. Engl. J. Med..

[113] Perl A.E., Martinelli G., Cortes J.E., Neubauer A., Berman E., Paolini S., Montesinos P., Baer M.R., Larson R.A., Ustun C. (2019). Gilteritinib or Chemotherapy for Relapsed or Refractory FLT3-Mutated AML. N. Engl. J. Med..

[114] Cortes J., Perl A.E., Döhner H., Kantarjian H., Martinelli G., Kovacsovics T., Rousselot P., Steffen B., Dombret H., Estey E. (2018). Quizartinib, an FLT3 inhibitor, as monotherapy in patients with relapsed or refractory acute myeloid leukaemia: An open-label, multicentre, single-arm, phase 2 trial. Lancet Oncol..

[115] Erba H.P., Montesinos P., Kim H.J., Patkowska E., Vrhovac R., Žák P., Wang P.N., Mitov T., Hanyok J., Kamel Y.M. (2023). Quizartinib plus chemotherapy in newly diagnosed patients with FLT3-internal-tandem-duplication-positive acute myeloid leukaemia (QuANTUM-First): A randomised, double-blind, placebo-controlled, phase 3 trial. Lancet.

[116] Xu Q., He S., Yu L. (2021). Clinical Benefits and Safety of FMS-Like Tyrosine Kinase 3 Inhibitors in Various Treatment Stages of Acute Myeloid Leukemia: A Systematic Review, Meta-Analysis, and Network Meta-Analysis. Front. Oncol..

[117] Bazinet A., Bataller A., Kadia T., Daver N., Short N.J., Yilmaz M., Sasaki K., DiNardo C.D., Borthakur G.M., Issa G. (2025). A retrospective study of outcomes across time and treatment regimens in newly diagnosed, FMS-like tyrosine kinase 3 (FLT3)-mutated acute myeloid leukemia. Cancer.

[118] Sato T., Yang X., Knapper S., White P., Smith B.D., Galkin S., Small D., Burnett A., Levis M. (2011). FLT3 ligand impedes the efficacy of FLT3 inhibitors in vitro and in vivo. Blood.

[119] Zhou J., Bi C., Janakakumara J.V., Liu S.C., Chng W.J., Tay K.G., Poon L.F., Xie Z., Palaniyandi S., Yu H. (2009). Enhanced activation of STAT pathways and overexpression of survivin confer resistance to FLT3 inhibitors and could be therapeutic targets in AML. Blood.

[120] Chatterjee A., Ghosh J., Ramdas B., Mali R.S., Martin H., Kobayashi M., Vemula S., Canela V.H., Waskow E.R., Visconte V. (2014). Regulation of Stat5 by FAK and PAK1 in Oncogenic FLT3- and KIT-Driven Leukemogenesis. Cell Rep..

[121] Traer E., Martinez J., Javidi-Sharifi N., Agarwal A., Dunlap J., English I., Kovacsovics T., Tyner J.W., Wong M., Druker B.J. (2016). FGF2 from Marrow Microenvironment Promotes Resistance to FLT3 Inhibitors in Acute Myeloid Leukemia. Cancer Res..

[122] Javidi-Sharifi N., Martinez J., English I., Joshi S.K., Scopim-Ribeiro R., Viola S.K., Edwards D.K., Agarwal A., Lopez C., Jorgens D. (2019). FGF2-FGFR1 signaling regulates release of Leukemia-Protective exosomes from bone marrow stromal cells. Elife.

[123] Joshi S.K., Nechiporuk T., Bottomly D., Piehowski P.D., Reisz J.A., Pittsenbarger J., Kaempf A., Gosline S.J.C., Wang Y.T., Hansen J.R. (2021). The AML microenvironment catalyzes a stepwise evolution to gilteritinib resistance. Cancer Cell.

[124] Khouqeer A., Cartailler J., Kim C., Dickerson K.M., Potts C.R., Gilbert A.E., Welner R.S., Ferrell P.B. (2025). IFNγ signaling drives resistance to FLT3 inhibition in acute myeloid leukemia. iScience.

[125] Rombouts E.J., Pavic B., Löwenberg B., Ploemacher R.E. (2004). Relation between CXCR-4 expression, Flt3 mutations, and unfavorable prognosis of adult acute myeloid leukemia. Blood.

[126] Ladikou E.E., Chevassut T., Pepper C.J., Pepper A.G. (2020). Dissecting the role of the CXCL12/CXCR4 axis in acute myeloid leukaemia. Br. J. Haematol..

[127] Fukuda S., Broxmeyer H.E., Pelus L.M. (2005). Flt3 ligand and the Flt3 receptor regulate hematopoietic cell migration by modulating the SDF-1alpha(CXCL12)/CXCR4 axis. Blood.

[128] Chang Y.T., Hernandez D., Alonso S., Gao M., Su M., Ghiaur G., Levis M.J., Jones R.J. (2019). Role of CYP3A4 in bone marrow microenvironment-mediated protection of FLT3/ITD AML from tyrosine kinase inhibitors. Blood Adv..

[129] Eriksson J., Zheng S., Popa M., Bao J., Dai J., Wang W., McCormack E., Vähärautio A., Tang J. (2025). Single-Cell Lineage Tracing Uncovers Resistance Signatures and Sensitizing Strategies to FLT3 Inhibitors in Acute Myeloid Leukemia. Cancer Res.

[130] Zhou Q., Li Z., Zhao P., Guan Y., Chu H., Xi Y. (2025). FLT3 inhibition upregulates OCT4/NANOG to promote maintenance and TKI resistance of FLT3-ITD+ acute myeloid leukemia. Oncogenesis.

[131] Smith C.C., Wang Q., Chin C.S., Salerno S., Damon L.E., Levis M.J., Perl A.E., Travers K.J., Wang S., Hunt J.P. (2012). Validation of ITD mutations in FLT3 as a therapeutic target in human acute myeloid leukaemia. Nature.

[132] Smith C.C., Lin K., Stecula A., Sali A., Shah N.P. (2015). FLT3 D835 mutations confer differential resistance to type II FLT3 inhibitors. Leukemia.

[133] Smith C.C., Paguirigan A., Jeschke G.R., Lin K.C., Massi E., Tarver T., Chin C.S., Asthana S., Olshen A., Travers K.J. (2017). Heterogeneous resistance to quizartinib in acute myeloid leukemia revealed by single-cell analysis. Blood.

[134] Heidel F., Solem F.K., Breitenbuecher F., Lipka D.B., Kasper S., Thiede M.H., Brandts C., Serve H., Roesel J., Giles F. (2006). Clinical resistance to the kinase inhibitor PKC412 in acute myeloid leukemia by mutation of Asn-676 in the FLT3 tyrosine kinase domain. Blood.

[135] Zhang H., Savage S., Schultz A.R., Bottomly D., White L., Segerdell E., Wilmot B., McWeeney S.K., Eide C.A., Nechiporuk T. (2019). Clinical resistance to crenolanib in acute myeloid leukemia due to diverse molecular mechanisms. Nat. Commun..

[136] Cools J., Mentens N., Furet P., Fabbro D., Clark J.J., Griffin J.D., Marynen P., Gilliland G. (2004). Prediction of resistance to small molecule FLT3 inhibitors: Implications for molecularly targeted therapy of acute leukemia. Cancer Res..

[137] Verma S., Singh A., Kumari A., Pandey B., Jamal S., Goyal S., Sinha S., Grover A. (2018). Insight into the inhibitor discrimination by FLT3 F691L. Chem. Biol. Drug Des..

[138] McMahon C.M., Canaani J., Rea B., Sargent R.L., Morissette J.J.D., Lieberman D.B., Watt C., Schwartz G.W., Faryabi R.B., Ferng T.T. (2017). Mechanisms of Acquired Resistance to Gilteritinib Therapy in Relapsed and Refractory FLT3-Mutated Acute Myeloid Leukemia. Blood.

[139] Chen F., Ishikawa Y., Akashi A., Naoe T., Kiyoi H. (2016). Co-expression of wild-type FLT3 attenuates the inhibitory effect of FLT3 inhibitor on FLT3 mutated leukemia cells. Oncotarget.

[140] Rummelt C., Gorantla S.P., Meggendorfer M., Charlet A., Endres C., Döhner K., Heidel F.H., Fischer T., Haferlach T., Duyster J. (2021). Activating JAK-mutations confer resistance to FLT3 kinase inhibitors in FLT3-ITD positive AML in vitro and in vivo. Leukemia.

[141] Chen P., Levis M., Brown P., Kim K.T., Allebach J., Small D. (2005). FLT3/ITD mutation signaling includes suppression of SHP-1. J. Biol. Chem..

[142] Park I.K., Mundy-Bosse B., Whitman S.P., Zhang X., Warner S.L., Bearss D.J., Blum W., Marcucci G., Caligiuri M.A. (2015). Receptor tyrosine kinase Axl is required for resistance of leukemic cells to FLT3-targeted therapy in acute myeloid leukemia. Leukemia.

[143] Kapoor S., Natarajan K., Baldwin P.R., Doshi K.A., Lapidus R.G., Mathias T.J., Scarpa M., Trotta R., Davila E., Kraus M. (2018). Concurrent Inhibition of Pim and FLT3 Kinases Enhances Apoptosis of FLT3-ITD Acute Myeloid Leukemia Cells through Increased Mcl-1 Proteasomal Degradation. Clin. Cancer Res..

[144] Long J., Jia M.Y., Fang W.Y., Chen X.J., Mu L.L., Wang Z.Y., Shen Y., Xiang R.F., Wang L.N., Wang L. (2020). FLT3 inhibition upregulates HDAC8 via FOXO to inactivate p53 and promote maintenance of FLT3-ITD+ acute myeloid leukemia. Blood.

[145] Puissant A., Fenouille N., Alexe G., Pikman Y., Bassil C.F., Mehta S., Du J., Kazi J.U., Luciano F., Rönnstrand L. (2014). SYK is a critical regulator of FLT3 in acute myeloid leukemia. Cancer Cell.

[146] Man C.H., Lam S.S., Sun M.K., Chow H.C., Gill H., Kwong Y.L., Leung A.Y. (2014). A novel tescalcin-sodium/hydrogen exchange axis underlying sorafenib resistance in FLT3-ITD+ AML. Blood.

[147] Hunter H.M., Pallis M., Seedhouse C.H., Grundy M., Gray C., Russell N.H. (2004). The expression of P-glycoprotein in AML cells with FLT3 internal tandem duplications is associated with reduced apoptosis in response to FLT3 inhibitors. Br. J. Haematol..

[148] Zhang Y., Wang P., Wang Y., Shen Y. (2023). Sitravatinib as a potent FLT3 inhibitor can overcome gilteritinib resistance in acute myeloid leukemia. Biomark. Res..

[149] Yang J., Zhang Y., Li Q., Wang P. (2025). Sitravatinib combined with venetoclax exerts effective synergy to eliminate acute myeloid leukemia cells with FLT3-ITD mutations. Transl. Oncol..

[150] Hu C., Zhang Y., Yang J., Xu Y., Deng T., Li Y., Xu S., Wang S., Wang P. (2024). Ningetinib, a novel FLT3 inhibitor, overcomes secondary drug resistance in acute myeloid leukemia. Cell Commun. Signal.

[151] Ferng T.T., Terada D., Ando M., Tarver T.C., Chaudhary F., Lin K.C., Logan A.C., Smith C.C. (2022). The Irreversible FLT3 Inhibitor FF-10101 Is Active Against a Diversity of FLT3 Inhibitor Resistance Mechanisms. Mol. Cancer Ther..

[152] Levis M., Perl A., Schiller G., Fathi A.T., Roboz G., Wang E.S., Altman J., Rajkhowa T., Ando M., Suzuki T. (2024). A phase 1 study of the irreversible FLT3 inhibitor FF-10101 in relapsed or refractory acute myeloid leukemia. Blood Adv..

[153] Errasti G., Delacroix T., Ghoshal K., Lee R., Ghosh A., Chakrabarti R. (2025). Novel potent and selective inhibitors targeting FLT3 for AML therapy. J. Clin. Oncol..

[154] Yu Z., Du J., Hui H., Kan S., Huo T., Zhao K., Wu T., Guo Q., Lu N. (2021). LT-171-861, a novel FLT3 inhibitor, shows excellent preclinical efficacy for the treatment of FLT3 mutant acute myeloid leukemia. Theranostics.

[155] Jeon J.Y., Buelow D.R., Garrison D.A., Niu M., Eisenmann E.D., Huang K.M., Zavorka Thomas M.E., Weber R.H., Whatcott C.J., Warner S.L. (2020). TP-0903 is active in models of drug-resistant acute myeloid leukemia. JCI Insight.

[156] Shi H., Wang M., Huang J., Quyang Q., Guo J., Wang Y., Mi Y., Wu H. (2023). CTS2016, a novel AXL/FLT3 inhibitor for targeting AML/MDS and solid tumors. Cancer Res..

[157] Liu Y., Wei J., Liu J., Ma W., Duan Y., Liu D. (2021). Novel AXL-targeted agents overcome FLT3 inhibitor resistance in FLT3-ITD+ acute myeloid leukemia cells. Oncol. Lett..

[158] Kasner M.T., Courtenay-Luck N., DiNardo C., Post S.M., Baratam P., Magrath G.N., Nakamura T., Fujii A., Prados S., Honda N. (2025). Anexelekto (Axl)/Mer Inhibitor Tamnorzatinib in Patients with Relapsed/Refractory Acute Myeloid Leukaemia: Results from a Phase I (Monotherapy) and Phase II (Combination with Venetoclax) Clinical Study. Acta Haematol.

[159] Xu S., Zhu Y., Meng J., Li C., Zhu Z., Wang C., Gu Y.C., Han L., Wen J., Tong M. (2023). 2-Aminopyrimidine derivatives as selective dual inhibitors of JAK2 and FLT3 for the treatment of acute myeloid leukemia. Bioorg Chem..

[160] Jeon J.Y., Zhao Q., Buelow D.R., Phelps M., Walker A.R., Mims A.S., Vasu S., Behbehani G., Blachly J., Blum W. (2020). Preclinical activity and a pilot phase I study of pacritinib, an oral JAK2/FLT3 inhibitor, and chemotherapy in FLT3-ITD-positive AML. Investig. New Drugs.

[161] Azhar M., Kincaid Z., Kesarwani M., Ahmed A., Wunderlich M., Latif T., Starczynowski D., Azam M. (2022). Momelotinib is a highly potent inhibitor of FLT3-mutant AML. Blood Adv..

[162] Morales M.L., Arenas A., Ortiz-Ruiz A., Leivas A., Rapado I., Rodriguez-Garcia A., Castro N., Zagorac I., Quintela-Fandino M., Gomez-Lopez G. (2019). MEK inhibition enhances the response to tyrosine kinase inhibitors in acute myeloid leukemia. Sci. Rep..

[163] Popescu B., Jones M.F., Piao M., Tran E., Koh A., Lomeli I., Peretz C.A.C., Murad N., Abelson S., Morales C. (2025). Multi-selective RAS(ON) Inhibition Targets Oncogenic RAS Mutations and Overcomes RAS/MAPK-Mediated Resistance to FLT3 and BCL2 Inhibitors in Acute Myeloid Leukemia. bioRxiv.

[164] Cho B.S., Zeng Z., Mu H., Wang Z., Konoplev S., McQueen T., Protopopova M., Cortes J., Marszalek J.R., Peng S.B. (2015). Antileukemia activity of the novel peptidic CXCR4 antagonist LY2510924 as monotherapy and in combination with chemotherapy. Blood.

[165] Zhang W., Chang K.H., Basyal M., Jia Y., Ostermann L.B., Fogler W.E., Magnani J.L., Zal M.A., Zal T., Andreeff M. (2020). Combined Blockage of E-Selectin and CXCR4 (GMI-1359) Enhances Anti-Leukemia Effect of FLT3 Inhibition (Sorafenib) and Protects Hematopoiesis in Pre-Clinical AML Models. Blood.

[166] Zhang W., Li L., Muftuoglu M., Basyal M., Togashi N., Iwanaga K., Tanzawa F., Numata M., Bixby D.L., Erba H.P. (2025). Synergistic Activity of Combined FLT3-ITD and MDM2 Inhibition with Quizartinib and Milademetan in FLT3-ITD Mutant/TP53 Wild-type Acute Myeloid Leukemias. Clin. Cancer Res..

[167] Uras I.Z., Walter G.J., Scheicher R., Bellutti F., Prchal-Murphy M., Tigan A.S., Valent P., Heidel F.H., Kubicek S., Scholl C. (2016). Palbociclib treatment of FLT3-ITD+ AML cells uncovers a kinase-dependent transcriptional regulation of FLT3 and PIM1 by CDK6. Blood.

[168] Sun C.Y., Talukder M., Cao D., Chen C.W. (2022). Gilteritinib Enhances Anti-Tumor Efficacy of CDK4/6 Inhibitor, Abemaciclib in Lung Cancer Cells. Front. Pharmacol..

[169] Peysin C., Nazha A., Wei W., Gerds A., Chen X., Carraway H., Singh A., Advani A., Ali M.M., Molina J. (2025). Final results of a phase I/II trial of palbociclib and CPX-351 in patients with Acute Myeloid Leukemia. Blood.

[170] Smith C.C., Levis M.J., Frankfurt O., Pagel J.M., Roboz G.J., Stone R.M., Wang E.S., Severson P.L., West B.L., Le M.H. (2020). A phase 1/2 study of the oral FLT3 inhibitor pexidartinib in relapsed/refractory FLT3-ITD-mutant acute myeloid leukemia. Blood Adv..

[171] Wang P., Zhang Y., Xiang R., Yang J., Xu Y., Deng T., Zhou W., Wang C., Xiao X., Wang S. (2024). Foretinib Is Effective in Acute Myeloid Leukemia by Inhibiting FLT3 and Overcoming Secondary Mutations That Drive Resistance to Quizartinib and Gilteritinib. Cancer Res..

[172] Daver N., Kyoo Lee K., Choi Y., Jonas B.A., Arellano M., Koller P.B., Jung C.W., Sohn S.K., Fathi A.T., Lee J.O. (2023). Tuspetinib Myeloid Kinase Inhibitor Safety and Efficacy As Monotherapy and Combined with Venetoclax in Phase 1/2 Trial of Patients with Relapsed or Refractory (R/R) Acute Myeloid Leukemia (AML). Blood.

[173] Rice W.G., Howell S.B., Zhang H., Rastgoo N., Local A., Kurtz S.E., Lo P., Bottomly D., Wilmot B., McWeeney S.K. (2022). Luxeptinib (CG-806) Targets FLT3 and Clusters of Kinases Operative in Acute Myeloid Leukemia. Mol. Cancer Ther..

[174] Moore A.S., Faisal A., Mak G.W.Y., Miraki-Moud F., Bavetsias V., Valenti M., Box G., Hallsworth A., de Haven Brandon A., Xavier C.P.R. (2020). Quizartinib-resistant FLT3-ITD acute myeloid leukemia cells are sensitive to the FLT3-Aurora kinase inhibitor CCT241736. Blood Adv..

[175] Wang P., Xiao X., Zhang Y., Zhang B., Li D., Liu M., Xie X., Liu C., Liu P., Ren R. (2021). Adual inhibitor overcomes drug-resistant FLT3-ITDacute myeloid leukemia. J. Hematol. Oncol..

[176] Cortes J., Tamura K., DeAngelo D.J., de Bono J., Lorente D., Minden M., Uy G.L., Kantarjian H., Chen L.S., Gandhi V. (2018). Phase I studies of AZD1208, a proviral integration Moloney virus kinase inhibitor in solid and haematological cancers. Br. J. Cancer.

[177] Yao M., Yan W., Wang Y., Zhao Y., Xu X., Chen Y., Yu C., Li Y., Jiang H., Shen J. (2025). IHCH9033, a novel class I HDAC inhibitor, synergizes with FLT3 inhibitor and rescues quizartinib resistance in FLT3-ITD AML via enhancing DNA damage response. Exp. Hematol. Oncol..

[178] Chang Y., Li X., Zhou Y., Yang X., Zhao W., Fang H., Hou X. (2024). Simultaneous inhibition of FLT3 and HDAC by novel 6-ethylpyrazine-2-Carboxamide derivatives provides therapeutic advantages in acute myelocytic leukemia. Eur. J. Med. Chem..

[179] Zhang T., Li Y., Liao W., Mou Y., Zhan X., Hu Q., Zhao Z., Xiong D. (2025). Decursin induces FLT3-ITD acute myeloid leukemia cell apoptosis by increasing the expression of the ubiquitin-conjugase UBE2L6. Cell Commun. Signal.

[180] Sun X., Li Y., Du J., Liu F., Wu C., Xiao W., Yu G., Chen X., Gale R.P., Zeng H. (2025). Targeting ceramide transfer protein sensitizes AML to FLT3 inhibitors via a GRP78-ATF6-CHOP axis. Nat. Commun..

[181] Chatterjee A., Mustafa Ali M.K., Bailey C.M., Liu Y., Small D., Smith C.C., Traer E., Wang Y., Silvestri G., Baer M.R. (2025). Sphingosine-1-phosphate receptor modulators resensitize FLT3-ITD acute myeloid leukemia cells with NRAS mutations to FLT3 inhibitors. bioRxiv.

[182] Fujii-Hanamoto A., Tanaka H., Fujimoto K., Haeno T., Miyake Y., Fujiwara R., Kumode T., Serizawa K., Morita Y., Hanamoto H. (2025). Antipsychotic Chlorpromazine Suppresses STAT5 Signaling, Overcomes Resistance Mediated by the Gatekeeper Mutation FLT3-ITD/F691L, and Synergizes with Quizartinib in FLT3-ITD-Positive Cells. Curr. Issues Mol. Biol..

[183] Zhang T., Wei D., Zhan Y., Long Z., Lu T., Zhao P., Gao R., Kang Q., Zhang L., Liu M. (2025). Heme oxygenase 1 confers gilteritinib resistance in FLT3-ITD acute myeloid leukemia in a STAT6-dependent manner. Cancer Cell Int..

[184] Sieberer H., Luciano M., Amend D., Blöchl C., Eglseer A., Steinkellner A., Rieser S., Andosch A., Steiner P., Hummer L. (2025). Inhibition of NLRP3 enhances pro-apoptotic effects of FLT3 inhibition in AML. Cell Commun. Signal.

[185] Rohrbacher L., Nixdorf D., Stadler H., Brauchle B., Märkl F., Gottschlich A., Hoffmann G.V., Philipp N., Hänel G., Kirmaier M.E. (2026). FLT3-directed BiTE Molecules versus CAR T Cells in AML: Co-stimulatory Signals Mitigate T-Cell Exhaustion. Blood Adv..

[186] Pedersen M.G., Møller B.K., Bak R.O. (2022). Recent Advances in the Development of Anti-FLT3 CAR T-Cell Therapies for Treatment of AML. Biomedicines.

[187] Abdul-Hay M., Vale C., Chan O., Chandhok N., DiNardo C., Schiller G., Wang E., Hong S., Bachiashvili K., Chaudhry M. (2025). Preliminary anti-leukemia activity from A phase 1 study of cln-049, a novel anti-FLT3 x anti-CD3 bispecific T-cell engager, in Relapsed/Refractory (R/R) Acute Myeloid Leukemia (AML) and myelodysplastic syndrome (MDS). Blood.

[188] https://www.onclive.com/view/cln-049-nets-fda-fast-track-designation-in-relapsed-refractory-aml.

[189] Reitman Z.J., Yan H. (2010). Isocitrate dehydrogenase 1 and 2 mutations in cancer: Alterations at a crossroads of cellular metabolism. J. Natl. Cancer Inst..

[190] Xu W., Yang H., Liu Y., Yang Y., Wang P., Kim S.H., Ito S., Yang C., Wang P., Xiao M.T. (2011). Oncometabolite 2-hydroxyglutarate is a competitive inhibitor of α-ketoglutarate-dependent dioxygenases. Cancer Cell.

[191] Oh S., Shin S., Janknecht R. (2019). The small members of the JMJD protein family: Enzymatic jewels or jinxes?. Biochim. Biophys. Acta Rev. Cancer.

[192] Paschka P., Schlenk R.F., Gaidzik V.I., Habdank M., Krönke J., Bullinger L., Späth D., Kayser S., Zucknick M., Götze K. (2010). IDH1 and IDH2 mutations are frequent genetic alterations in acute myeloid leukemia and confer adverse prognosis in cytogenetically normal acute myeloid leukemia with NPM1 mutation without FLT3 internal tandem duplication. J. Clin. Oncol..

[193] Marcucci G., Maharry K., Wu Y.Z., Radmacher M.D., Mrózek K., Margeson D., Holland K.B., Whitman S.P., Becker H., Schwind S. (2010). IDH1 and IDH2 gene mutations identify novel molecular subsets within de novo cytogenetically normal acute myeloid leukemia: A Cancer and Leukemia Group B study. J. Clin. Oncol..

[194] Messina M., Piciocchi A., Ottone T., Paolini S., Papayannidis C., Lessi F., Fracchiolla N.S., Forghieri F., Candoni A., Mengarelli A. (2022). Prevalence and Prognostic Role of IDH Mutations in Acute Myeloid Leukemia: Results of the GIMEMA AML1516 Protocol. Cancers.

[195] Raimondi V., Ciotti G., Gottardi M., Ciccarese F. (2022). 2-Hydroxyglutarate in Acute Myeloid Leukemia: A Journey from Pathogenesis to Therapies. Biomedicines.

[196] Ježek P. (2020). 2-Hydroxyglutarate in Cancer Cells. Antioxid. Redox Signal.

[197] Chen F., Bian K., Tang Q., Fedeles B.I., Singh V., Humulock Z.T., Essigmann J.M., Li D. (2017). Oncometabolites d- and l-2-Hydroxyglutarate Inhibit the AlkB Family DNA Repair Enzymes under Physiological Conditions. Chem. Res. Toxicol..

[198] Desai P., Mencia-Trinchant N., Savenkov O., Simon M.S., Cheang G., Lee S., Samuel M., Ritchie E.K., Guzman M.L., Ballman K.V. (2018). Somatic mutations precede acute myeloid leukemia years before diagnosis. Nat. Med..

[199] Tuval A., Shlush L.I. (2019). Evolutionary trajectory of leukemic clones and its clinical implications. Haematologica.

[200] Deng G., Shen J., Yin M., McManus J., Mathieu M., Gee P., He T., Shi C., Bedel O., McLean L.R. (2015). Selective inhibition of mutant isocitrate dehydrogenase 1 (IDH1) via disruption of a metal binding network by an allosteric small molecule. J. Biol. Chem..

[201] Stein E.M., DiNardo C.D., Pollyea D.A., Fathi A.T., Roboz G.J., Altman J.K., Stone R.M., DeAngelo D.J., Levine R.L., Flinn I.W. (2017). Enasidenib in mutant IDH2 relapsed or refractory acute myeloid leukemia. Blood.

[202] https://www.fda.gov/drugs/resources-information-approved-drugs/fda-approves-ivosidenib-relapsed-or-refractory-acute-myeloid-leukemia.

[203] https://www.fda.gov/drugs/resources-information-approved-drugs/fda-approves-ivosidenib-combination-azacitidine-newly-diagnosed-acute-myeloid-leukemia.

[204] https://www.fda.gov/drugs/resources-information-approved-drugs/fda-approves-olutasidenib-relapsed-or-refractory-acute-myeloid-leukemia-susceptible-idh1-mutation.

[205] https://www.fda.gov/drugs/resources-information-approved-drugs/fda-granted-regular-approval-enasidenib-treatment-relapsed-or-refractory-aml.

[206] Chen X., Xing H., Xie X., Kou L., Li J., Li Y. (2023). Efficacy and safety of FDA-approved IDH inhibitors in the treatment of IDH mutated acute myeloid leukemia: A systematic review and meta-analysis. Clin. Epigenetics.

[207] DiNardo C.D., Stein E.M., de Botton S., Roboz G.J., Altman J.K., Mims A.S., Swords R., Collins R.H., Mannis G.N., Pollyea D.A. (2018). Durable Remissions with Ivosidenib in IDH1-Mutated Relapsed or Refractory AML. N. Engl. J. Med..

[208] Choe S., Wang H., DiNardo C.D., Stein E.M., de Botton S., Roboz G.J., Altman J.K., Mims A.S., Watts J.M., Pollyea D.A. (2020). Molecular mechanisms mediating relapse following ivosidenib monotherapy in IDH1-mutant relapsed or refractory AML. Blood Adv..

[209] Amatangelo M.D., Quek L., Shih A., Stein E.M., Roshal M., David M.D., Marteyn B., Farnoud N.R., de Botton S., Bernard O.A. (2017). Enasidenib induces acute myeloid leukemia cell differentiation to promote clinical response. Blood.

[210] Liu A., Cathelin S., Ayyathan D., Yang Y., Aboualizadeh F., Abow A., Basi G., Li L., Dai D.L., Maher A. (2026). Single-cell proteogenomic analysis of clonal evolution in PDX models of AML treated with IDH inhibitors. Blood Neoplasia.

[211] Wang F., Morita K., DiNardo C.D., Furudate K., Tanaka T., Yan Y., Patel K.P., MacBeth K.J., Wu B., Liu G. (2021). Leukemia stemness and co-occurring mutations drive resistance to IDH inhibitors in acute myeloid leukemia. Nat. Commun..

[212] Zhan T., Rindtorff N., Boutros M. (2017). Wnt signaling in cancer. Oncogene.

[213] Salik B., Yi H., Hassan N., Santiappillai N., Vick B., Connerty P., Duly A., Trahair T., Woo A.J., Beck D. (2020). Targeting RSPO3-LGR4 Signaling for Leukemia Stem Cell Eradication in Acute Myeloid Leukemia. Cancer Cell.

[214] Quek L., David M.D., Kennedy A., Metzner M., Amatangelo M., Shih A., Stoilova B., Quivoron C., Heiblig M., Willekens C. (2018). Clonal heterogeneity of acute myeloid leukemia treated with the IDH2 inhibitor enasidenib. Nat. Med..

[215] Harding J.J., Lowery M.A., Shih A.H., Schvartzman J.M., Hou S., Famulare C., Patel M., Roshal M., Do R.K., Zehir A. (2018). Isoform Switching as a Mechanism of Acquired Resistance to Mutant Isocitrate Dehydrogenase Inhibition. Cancer Discov..

[216] Intlekofer A.M., Shih A.H., Wang B., Nazir A., Rustenburg A.S., Albanese S.K., Patel M., Famulare C., Correa F.M., Takemoto N. (2018). Acquired resistance to IDH inhibition through trans or cis dimer-interface mutations. Nature.

[217] Lindahl E., Arvidsson E., Friedman R. (2024). Trans vs. cis: A computational study of enasidenib resistance due to IDH2 mutations. Phys. Chem. Chem. Phys..

[218] Reinbold R., Hvinden I.C., Rabe P., Herold R.A., Finch A., Wood J., Morgan M., Staudt M., Clifton I.J., Armstrong F.A. (2022). Resistance to the isocitrate dehydrogenase 1 mutant inhibitor ivosidenib can be overcome by alternative dimer-interface binding inhibitors. Nat. Commun..

[219] Avellaneda Matteo D., Wells G.A., Luna L.A., Grunseth A.J., Zagnitko O., Scott D.A., Hoang A., Luthra A., Swairjo M.A., Schiffer J.M. (2018). Inhibitor potency varies widely among tumor-relevant human isocitrate dehydrogenase 1 mutants. Biochem. J..

[220] Stuani L., Sabatier M., Saland E., Cognet G., Poupin N., Bosc C., Castelli F.A., Gales L., Turtoi E., Montersino C. (2021). Mitochondrial metabolism supports resistance to IDH mutant inhibitors in acute myeloid leukemia. J. Exp. Med..

[221] Lyu J., Liu Y., Liu N., Vu H.S., Cai F., Cao H., Kaphle P., Wu Z., Botten G.A., Zhang Y. (2025). CD44-mediated metabolic rewiring is a targetable dependency of IDH-mutant leukemia. Blood.

[222] Montesinos P., Marchione D.M., Recher C., Heuser M., Vives S., Zarzycka E., Wang J., Riva M., Calado R.T., Schuh A.C. (2025). Long-term results from the AGILE study of azacitidine plus ivosidenib vs placebo in newly diagnosed IDH1-mutated AML. Blood Adv..

[223] DiNardo C.D., Schuh A.C., Stein E.M., Montesinos P., Wei A.H., de Botton S., Zeidan A.M., Fathi A.T., Kantarjian H.M., Bennett J.M. (2021). Enasidenib plus azacitidine versus azacitidine alone in patients with newly diagnosed, mutant-IDH2 acute myeloid leukaemia (AG221-AML-005): A single-arm, phase 1b and randomised, phase 2 trial. Lancet Oncol..

[224] MacBeth K.J., Chopra V.S., Tang L., Zheng B., Avanzino B., See W.L., Schwickart M., Figueroa M.E., Quek L., DiMartino J.F. (2021). Combination of azacitidine and enasidenib enhances leukemic cell differentiation and cooperatively hypomethylates DNA. Exp. Hematol..

[225] Gruber E., So J., Lewis A.C., Franich R., Cole R., Martelotto L.G., Rogers A.J., Vidacs E., Fraser P., Stanley K. (2022). Inhibition of mutant IDH1 promotes cycling of acute myeloid leukemia stem cells. Cell Rep..

[226] Chaturvedi A., Gupta C., Gabdoulline R., Borchert N.M., Goparaju R., Kaulfuss S., Görlich K., Schottmann R., Othman B., Welzenbach J. (2021). Synergistic activity of IDH1 inhibitor BAY1436032 with azacitidine in IDH1 mutant acute myeloid leukemia. Haematologica.

[227] Richard-Carpentier G., Gupta G., Cameron C., Cathelin S., Bankar A., Davidson M.B., Gupta V., Maze D.C., Minden M.D., Murphy T. (2023). Final Results of the Phase Ib/II Study Evaluating Enasidenib in Combination with Venetoclax in Patients with IDH2-Mutated Relapsed/Refractory Myeloid Malignancies. Blood.

[228] Lachowiez C.A., Loghavi S., Zeng Z., Tanaka T., Kim Y.J., Uryu H., Turkalj S., Jakobsen N.A., Luskin M.R., Duose D.Y. (2023). A Phase Ib/II Study of Ivosidenib with Venetoclax ± Azacitidine in IDH1-Mutated Myeloid Malignancies. Blood Cancer Discov..

[229] Libura M., Bialopiotrowicz E., Giebel S., Wierzbowska A., Roboz G.J., Piatkowska-Jakubas B., Pawelczyk M., Gorniak P., Borg K., Wojtas M. (2021). IDH2 mutations in patients with normal karyotype AML predict favorable responses to daunorubicin, cytarabine and cladribine regimen. Sci. Rep..

[230] Morell A., Budagaga Y., Vagiannis D., Zhang Y., Laštovičková L., Novotná E., Haddad A., Haddad M., Portillo R., Hofman J. (2022). Isocitrate dehydrogenase 2 inhibitor enasidenib synergizes daunorubicin cytotoxicity by targeting aldo-keto reductase 1C3 and ATP-binding cassette transporters. Arch. Toxicol..

[231] Stein E., DiNardo C., Fathi A., Mims A., Pratz K., Savona M., Stein A.S., Stone R., Winer E., Seet C. (2025). Updated response and safety analyses from a Phase 1 study of ivosidenib combined with intensive chemotherapy in patients with newly diagnosed (ND) Acute Myeloid Leukemia with isocitrate dehydrogenase (IDH)1 mutation. Blood.

[232] DiNardo C.D., De Botton S., Pollyea D.A., Stone R.M., Altman J.K., Fathi A.T., Limsakun T., Liang M., Choe S., Hossain M. (2023). Safety, efficacy, and PK/PD of vorasidenib in previously treated patients with mIDH1/2 hematologic malignancies: A phase 1 study. Am. J. Hematol..

[233] DiNardo C.D., Montesinos P., Benajiba L., Triguero A., Recher C., Schuh A.C., Heiblig M., Bajel A., Pigneux A., Alonso-Domiguez J.M. (2023). A first-in-human phase 1 study of LY3410738, a covalent inhibitor of mutant IDH, in advanced myeloid malignancies. Cancer Res..

[234] Xiao K., Zhang Z., Wu Y., Li G., Chen J., Ren Y., Yang N., Zhou J., Zhang W., Wang J. (2025). Discovery of HMPL-306 (Ranosidenib), a New Potent and Selective Dual Inhibitor of Mutant IDH1 and 2 in Clinical Development for Cancer Treatment. ACS Med. Chem. Lett..

[235] Hu L., Wei X., Zhao W., Hu Y., Li J., Dong Y., Gong T., Zhang X., Xu Y., Zhang Y. (2025). HMPL-306 in relapsed or refractory IDH1- and/or IDH2-mutated acute myeloid leukemia: A phase 1 study. Med.

[236] Chaturvedi A., Goparaju R., Gupta C., Weder J., Klünemann T., Araujo Cruz M.M., Kloos A., Goerlich K., Schottmann R., Othman B. (2020). In vivo efficacy of mutant IDH1 inhibitor HMS-101 and structural resolution of distinct binding site. Leukemia.

[237] Li X., Song Y. (2020). Proteolysis-targeting chimera (PROTAC) for targeted protein degradation and cancer therapy. J. Hematol. Oncol..

[238] Dutta H., Jain N. (2025). Degrading mutant IDH1 employing a PROTAC-based approach impairs STAT3 activation. Arch. Biochem. Biophys..

[239] Hujber Z., Petővári G., Szoboszlai N., Dankó T., Nagy N., Kriston C., Krencz I., Paku S., Ozohanics O., Drahos L. (2017). Rapamycin (mTORC1 inhibitor) reduces the production of lactate and 2-hydroxyglutarate oncometabolites in IDH1 mutant fibrosarcoma cells. J. Exp. Clin. Cancer Res..

[240] Ball B.J., Zhang J., Afkhami M., Robbins M., Chang L., Humpal S., Porras V., Liu Y., Synold T.W., Farzinkhou S. (2024). Phase 1b Study of IDH Inhibition with Enasidenib and MEK Inhibition with Cobimetinib in Patients with Relapsed or Refractory Acute Myeloid Leukemia Who Have Co-Occurring IDH2 and RAS Signaling Gene Mutations. Blood.

[241] Martinelli G., Solomon S.R., Mukherjee S., Santoro A., Strickland S.A., Vives S., Ravandi F., Walter R.B., Cook R.J., Lech-Maranda E. (2026). Dual FLT3/PIM inhibitor dapolsertib in acute myeloid leukemia: Results from the phase 1/2 DIAMOND-01 trial. Blood Neoplasia.

[242] Gbyli R., Song Y., Liu W., Gao Y., Biancon G., Chandhok N.S., Wang X., Fu X., Patel A., Sundaram R. (2022). In vivo anti-tumor effect of PARP inhibition in IDH1/2 mutant MDS/AML resistant to targeted inhibitors of mutant IDH1/2. Leukemia.

[243] Thomas D., Wu M., Nakauchi Y., Zheng M., Thompson-Peach C.A.L., Lim K., Landberg N., Köhnke T., Robinson N., Kaur S. (2023). Dysregulated Lipid Synthesis by Oncogenic IDH1 Mutation Is a Targetable Synthetic Lethal Vulnerability. Cancer Discov..

[244] Candoni A., Coppola G. (2024). A 2024 Update on Menin Inhibitors. A New Class of Target Agents against KMT2A-Rearranged and NPM1-Mutated Acute Myeloid Leukemia. Hematol. Rep..

[245] https://www.fda.gov/drugs/resources-information-approved-drugs/fda-approves-revumenib-relapsed-or-refractory-acute-leukemia-kmt2a-translocation.

[246] https://www.fda.gov/drugs/resources-information-approved-drugs/fda-approves-revumenib-relapsed-or-refractory-acute-myeloid-leukemia-susceptible-npm1-mutation.

[247] https://www.fda.gov/drugs/resources-information-approved-drugs/fda-approves-ziftomenib-relapsed-or-refractory-acute-myeloid-leukemia-npm1-mutation.

[248] Döhner H., Schuh A., Recher C., O’Nions J., Aldoss I., Alfonso-Pierola A., Allred A., Alonso-Dominguez J.M., Barreyro L., Bories P. (2025). Bleximenib in combination with intensive chemotherapy: A phase 1b study in newly diagnosed Acute Myeloid Leukemia with KMT2A or NPM1 alterations. Blood.

[249] Zeidner J.F., Yuda J., Watts J.M., Levis M.J., Erba H.P., Fukushima K., Shima T., Palmisiano N.D., Wang E.S., Borate U. (2024). Phase 1 Results: First-in-Human Phase 1/2 Study of the Menin-MLL Inhibitor Enzomenib (DSP-5336) in Patients with Relapsed or Refractory Acute Leukemia. Blood.

[250] Senapati J., Konopleva M., Issa G.C., Jabbour E., Kadia T., DiNardo C., Borthakur G., Pemmaraju N., Short N.J., Yilmaz M. (2025). A phase 1/2 study of DS-1594 menin inhibitor in relapsed/refractory acute leukemias. J. Hematol. Oncol..

[251] Arellano M.L., Thirman M.J., DiPersio J.F., Heiblig M., Stein E.M., Schuh A.C., Žučenka A., de Botton S., Grove C.S., Mannis G.N. (2025). Menin inhibition with revumenib for NPM1-mutated relapsed or refractory acute myeloid leukemia: The AUGMENT-101 study. Blood.

[252] Issa G.C., Aldoss I., Thirman M.J., DiPersio J., Arellano M., Blachly J.S., Mannis G.N., Perl A., Dickens D.S., McMahon C.M. (2025). Menin Inhibition With Revumenib for KMT2A-Rearranged Relapsed or Refractory Acute Leukemia (AUGMENT-101). J. Clin. Oncol..

[253] Wang E.S., Issa G.C., Erba H.P., Altman J.K., Montesinos P., DeBotton S., Walter R.B., Pettit K., Savona M.R., Shah M.V. (2024). Ziftomenib in relapsed or refractory acute myeloid leukaemia (KOMET-001): A multicentre, open-label, multi-cohort, phase 1 trial. Lancet Oncol..

[254] Wang E.S., Montesinos P., Foran J., Erba H., Rodríguez-Arbolí E., Fedorov K., Heiblig M., Heidel F.H., Altman J.K., Baer M.R. (2025). Ziftomenib in Relapsed or Refractory NPM1-Mutated AML. J. Clin. Oncol..

[255] Perner F., Stein E.M., Wenge D.V., Singh S., Kim J., Apazidis A., Rahnamoun H., Anand D., Marinaccio C., Hatton C. (2023). MEN1 mutations mediate clinical resistance to menin inhibition. Nature.

[256] Ray J., Clegg B., Grembecka J., Cierpicki T. (2024). Drug-resistant menin variants retain high binding affinity and interactions with MLL1. J. Biol. Chem..

[257] Thakur R.K., Wang E.S. (2025). The promise of menin inhibitors: From approval to triplet regimens. Hematol. Am. Soc. Hematol. Educ. Program..

[258] Zhou X., Zhang L., Aryal S., Veasey V., Tajik A., Restelli C., Moreira S., Zhang P., Zhang Y., Hope K. (2024). Epigenetic regulation of noncanonical menin targets modulates menin inhibitor response in acute myeloid leukemia. Blood.

[259] Soto-Feliciano Y.M., Sánchez-Rivera F.J., Perner F., Barrows D.W., Kastenhuber E.R., Ho Y.J., Carroll T., Xiong Y., Anand D., Soshnev A.A. (2023). A Molecular Switch between Mammalian MLL Complexes Dictates Response to Menin-MLL Inhibition. Cancer Discov..

[260] Janssens D.H., Duran M., Otto D.J., Wu W., Xu Y., Kirkey D., Mullighan C.G., Yi J.S., Meshinchi S., Sarthy J.F. (2024). KMT2A oncoproteins induce epigenetic resistance to targeted therapies. bioRxiv.

[261] Perner F., Rahnamoun H., Wenge D.V., Xiong Y., Apazidis A., Anand D., Hatton C., Wen Y., Gu S., Liu X.S. (2023). Non-Genetic Resistance to Menin Inhibtion in AML is Reversible by Pertubation of KAT6A. HemaSphere.

[262] Chin K.K., Ball B.J., Abaza Y., Altman J.K., Thakur R.K., Ali M., Derkach A., Geyer M.B., Goldberg A.D., Haque T.Z. (2025). Outcomes of relapsed or refractory acute myeloid leukemia after menin inhibition failure. Blood Adv..

[263] Allen B., Savoy L., Ryabinin P., Bottomly D., Chen R., Goff B., Wang A., McWeeney S.K., Zhang H. (2024). Upregulation of HOXA3 by isoform-specific Wilms tumour 1 drives chemotherapy resistance in acute myeloid leukaemia. Br. J. Haematol..

[264] Mahdavi L., Alikarami F., Goodrow H., Lenard A., Riedel S.S., Libbrecht C., Bowser I., Tasian S.K., Falkenstein C.D., Manning B. (2026). Upfront menin-inhibitor resistance in multiply pretreated leukemias. Exp. Hematol..

[265] https://charmtx.com/charm-therapeutics-advances-a-next-generation-menin-inhibitor-as-its-first-clinical-candidate-for-acute-myeloid-leukaemia/.

[266] Ciaurro V., Sharlandjieva V., Skwarska A., Chahrour C., Baran N., Zeng Z., Ramage C., Daver N., Carter B.Z., Chaundhry S. (2025). Menin inhibitor DS-1594b drives differentiation and induces synergistic lethality in combination with venetoclax in acute myeloid leukemia cells with rearranged mixed-lineage leukemia and mutated nucleophosmin-1. Haematologica.

[267] Carter B.Z., Tao W., Mak P.Y., Ostermann L.B., Mak D., McGeehan G., Ordentlich P., Andreeff M. (2021). Menin inhibition decreases Bcl-2 and synergizes with venetoclax in NPM1/FLT3-mutated AML. Blood.

[268] Fathi A.T., Issa G.C., Wang E.S., Erba H., Altman J.K., Balasubramanian S.K., Roboz G.J., Schiller G.J., McMahon C.M., Palmisiano N.D. (2024). Ziftomenib Combined with Venetoclax/Azacitidine in Relapsed/Refractory NPM1-m or KMT2A-r Acute Myeloid Leukemia: Interim Phase 1a Results from KOMET-007. Blood.

[269] Zeidner J.F., Lin T.L., Welkie R.L., Curran E., Koenig K., Stock W., Madanat Y.F., Swords R., Baer M.R., Blum W. (2025). Azacitidine, Venetoclax, and Revumenib for Newly Diagnosed NPM1-Mutated or KMT2A-Rearranged AML. J. Clin. Oncol..

[270] Khazaei S., Nair S., Ostermann L., Mizuno H., Mak P.Y., Carter B., Boettcher S., Ma L., Garcia M., Cuglievan B. (2025). Protein degradation of MYC/GSPT1 combined with menin inhibition overcomes resistance to menin inhibition in KMT2A-rearranged Acute Myeloid Leukemia. Blood.

[271] Fiskus W., Piel J., Collins M., Hentemann M., Cuglievan B., Mill C.P., Birdwell C.E., Das K., Davis J.A., Hou H. (2024). BRG1/BRM inhibitor targets AML stem cells and exerts superior preclinical efficacy combined with BET or menin inhibitor. Blood.

[272] Zhu W., Ding Y., Huang W., Guo N., Ren Q., Wang N., Ma X. (2024). Synergistic effects of the KDM4Cinhibitor SD70 and the menin inhibitor MI-503 against MLL::AF9-driven acute myeloid leukaemia. Br. J. Haematol..

[273] Tayari M.M., Dos Santos H.G., Beckedorff F., Totiger T., Mookhtiar A., Kingham A.L., Lavezzo G.M., Martin G.M., Huang H.T., Karaca E. (2025). Co-targeting menin and LSD1 dismantles oncogenic programs and restores differentiation in MLL-rearranged AML. bioRxiv.

[274] Shi X., Li M., Liu Z., Tiessen J., Li Y., Zhou J., Zhu Y., Mahesula S., Ding Q., Tan L. (2025). Guanine nucleotide biosynthesis blockade impairs MLL complex formation and sensitizes leukemias to menin inhibition. Nat. Commun..

[275] Adriaanse F.R.S., Schneider P., Arentsen-Peters S.T.C.J.M., Fonseca A.M.N.D., Stutterheim J., Pieters R., Zwaan C.M., Stam R.W. (2024). Distinct Responses to Menin Inhibition and Synergy with DOT1L Inhibition in KMT2A-Rearranged Acute Lymphoblastic and Myeloid Leukemia. Int. J. Mol. Sci..

[276] Gordon S.J.V., Perner F., MacPherson L., Fennell K.A., Wenge D.V., Bourgeois W., Klaus T., Plenge T., Murat A., Petrovic J. (2025). Catalytic Inhibition of KAT6/KAT7 Enhances the Efficacy and Overcomes Primary and Acquired Resistance to Menin Inhibitors in MLL Leukemia. Cancer Discov..

[277] Fleischmann M., Bechwar J., Voigtländer D., Fischer M., Schnetzke U., Hochhaus A., Scholl S. (2024). Synergistic Effects of the RARalpha Agonist Tamibarotene and the Menin Inhibitor Revumenib in Acute Myeloid Leukemia Cells with KMT2A Rearrangement or NPM1 Mutation. Cancers.

[278] https://abstracts.texascancerconference.org/View/491.

